# From Biogas and Hydrogen to Microbial Protein Through Co-Cultivation of Methane and Hydrogen Oxidizing Bacteria

**DOI:** 10.3389/fbioe.2021.733753

**Published:** 2021-08-30

**Authors:** Frederiek-Maarten Kerckhof, Myrsini Sakarika, Marie Van Giel, Maarten Muys, Pieter Vermeir, Jo De Vrieze, Siegfried E. Vlaeminck, Korneel Rabaey, Nico Boon

**Affiliations:** ^1^ Center for Microbial Ecology and Technology, Faculty of Bioscience Engineering, Ghent University, Gent, Belgium; ^2^ Center for Advanced Process Technology for Urban Resource Recovery (CAPTURE), Gent, Belgium; ^3^ Research Group of Sustainable Energy, Air and Water Technology, Department of Bioscience Engineering, University of Antwerp, Antwerpen, Belgium; ^4^ Laboratory of Chemical Analysis, Department of Green Chemistry and Technology, Faculty of Bioscience Engineering, Ghent University, Ghent, Belgium

**Keywords:** single-cell protein (SCP), methanotroph communities, hydrogenotroph, hydrogen oxidizing bacteria, synthetic community, sustainable protein, protein quality

## Abstract

Increasing efforts are directed towards the development of sustainable alternative protein sources among which microbial protein (MP) is one of the most promising. Especially when waste streams are used as substrates, the case for MP could become environmentally favorable. The risks of using organic waste streams for MP production–the presence of pathogens or toxicants–can be mitigated by their anaerobic digestion and subsequent aerobic assimilation of the (filter-sterilized) biogas. Even though methane and hydrogen oxidizing bacteria (MOB and HOB) have been intensively studied for MP production, the potential benefits of their co-cultivation remain elusive. Here, we isolated a diverse group of novel HOB (that were capable of autotrophic metabolism), and co-cultured them with a defined set of MOB, which could be grown on a mixture of biogas and H_2_/O_2_. The combination of MOB and HOB, apart from the CH_4_ and CO_2_ contained in biogas, can also enable the valorization of the CO_2_ that results from the oxidation of methane by the MOB. Different MOB and HOB combinations were grown in serum vials to identify the best-performing ones. We observed synergistic effects on growth for several combinations, and in all combinations a co-culture consisting out of both HOB and MOB could be maintained during five days of cultivation. Relative to the axenic growth, five out of the ten co-cultures exhibited 1.1–3.8 times higher protein concentration and two combinations presented 2.4–6.1 times higher essential amino acid content. The MP produced in this study generally contained lower amounts of the essential amino acids histidine, lysine and threonine, compared to tofu and fishmeal. The most promising combination in terms of protein concentration and essential amino acid profile was *Methyloparacoccus murrelli* LMG 27482 with *Cupriavidus necator* LMG 1201. Microbial protein from *M. murrelli* and *C. necator* requires 27–67% less quantity than chicken, whole egg and tofu, while it only requires 15% more quantity than the amino acid-dense soybean to cover the needs of an average adult. In conclusion, while limitations still exist, the co-cultivation of MOB and HOB creates an alternative route for MP production leveraging safe and sustainably-produced gaseous substrates.

## Introduction

The world population is projected to increase to 9.6–12.3 billion people by 2,100 (Gerland et al., 2014). The parallel rising demand for nutritional food per person (Westhoek et al., 2011), coupled with the Westernization of diets, results in increasing protein consumption (Statovci et al., 2017). Protein is undoubtedly one of the most important constituents in the human and animal diet, and without it, the cells, tissues and the whole body cannot function properly (Wu, 2014). Safeguarding its supply can be a particularly difficult task, since the population increase coupled with the global dietary changes (Delgado, 2003) create a yawning gap between food demand and supply (FAO et al., 2019).

The global consumption of animal products is ever increasing (WHO, 2012), but their production through current agricultural practices results in detrimental environmental effects. For instance, agriculture is notorious for its water consumption and greenhouse gas emissions. About 92% of global freshwater use is attributed to agricultural practices, while nearly 7.1 Gt CO_2_-equivalents are globally emitted each year due to food production, representing 14.5% of the overall anthropogenic emissions (Gerber et al., 2013). The production of livestock is responsible for the occupation of about 70% of the available arable land (Hashempour-Baltork et al., 2020). Additionally, fishmeal-fed aquaculture is highly unsustainable since the production of edible fish results in the depletion of wild fish stocks, due to the fishmeal dependency as aquaculture feed (Naylor et al., 2000), although sustainable aquaculture feed alternatives do exist (e.g., Crab et al. (2012)). Coupled with the inefficiency of the production of animal products (Apaiah et al., 2006), these facts point out the need for a so-called “protein transition” (Aiking and de Boer, 2018).

Microbial protein (MP) or single-cell protein (SCP) could alleviate the environmental and socio-economic pressure caused by the limitations of conventional agriculture, because it can provide nutritional protein, based on recovered resources (Matassa et al., 2015). MP is the biomass of microbes, such as fungi, yeasts, microalgae and bacteria, which contain all the essential amino acids (EAA) and can replace conventional protein food/feed sources (Ravindra, 2000). A recent life cycle assessment (LCA) revealed the lower environmental impact of MP compared to the use of soybean meal as a feed ingredient (Spiller et al., 2020). The greatest example of commercial production of MP as a food ingredient is Quorn™ (Marlow Foods, United Kingdom), which consists of mycobacterial biomass (i.e., *Fusarium venenatum*), and it is available in multiple countries worldwide (Wiebe, 2004). Other commercially available examples are feed ingredients produced on natural gas, such as Feedkind® (Calysta, United States) and Uniprotein® (Unibio A/S, Denmark), as well as MP from H_2_/CO_2_ such as Proton™ (Deep Branch Biotechnology, United Kingdom), while other relevant products emerge (Pander et al., 2020).

There is a broad variety of substrates, metabolisms and microorganisms that can be used for the production of MP, each having its merits (Ritala et al., 2017; Linder, 2019). Methane oxidizing bacteria (MOB), also known as methanotrophs, use methane (CH_4_) as carbon and energy source. Hydrogen oxidizing bacteria (HOB), use hydrogen (H_2_) as an energy source and can use carbon dioxide (CO_2_) as a carbon source. Both MOB and HOB are biotechnologically interesting organisms, able to produce a variety of products, ranging from MP to biopolymers and high value compounds like pigments and ectoine (Davis et al., 1969; Strong et al., 2015).

**FIGURE 1:**
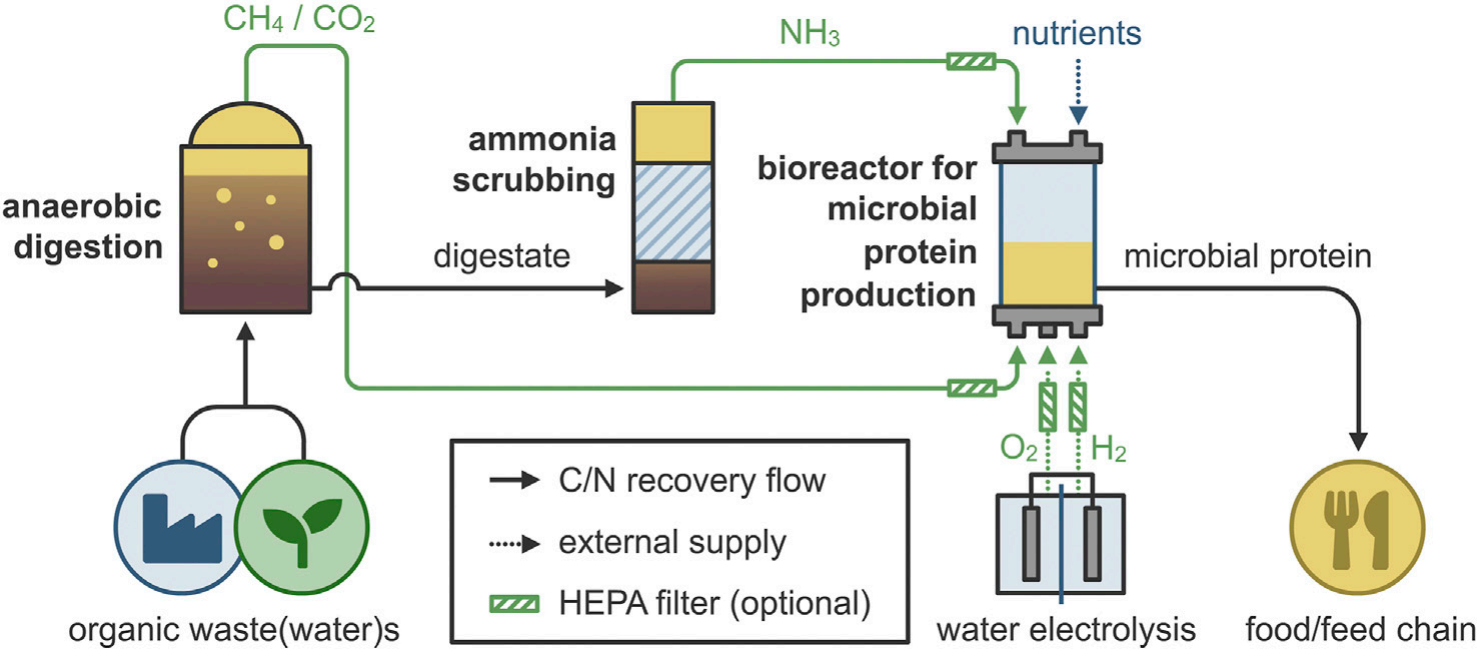
The gas clearance concept for microbial protein production. Organic waste is anaerobically digested to produce methane (CH_4_) and carbon dioxide (CO_2_), while ammonia (NH_3_) is recovered through stripping. Next, hydrogen (H_2_) and oxygen (O_2_) are produced from water electrolysis using renewable energy, and both are supplemented in the bioreactor for microbial protein production, along with the required nutrients. After the necessary downstream processing, the microbial protein can be used as food or feed (additive).

Even though MOB are already commercially produced as MP (in the Feedkind® and Uniprotein® processes listed above), their production relies on fossil-based resources. The natural gas used in this process can be substituted by biogas, produced via anaerobic digestion of waste streams (Van der Ha et al., 2012; Acosta et al., 2019; Verbeeck et al., 2021). These biogenically sourced CH_4_ and CO_2_ from secondary materials do not cause food competition since they do not directly depend on the food chain (side streams of the food industry and food waste can be used as feedstocks for anaerobic digestion), and they do not require arable land or potable water (IEA, 2020). The combination of MOB and HOB would enable the valorization of the CH_4_ and CO_2_ that is contained in raw biogas, as well as the CO_2_ that results from the oxidation of CH_4_, resulting in the “gas clearance” concept for MP production (Figure 1). The required H_2_ and O_2_ can be produced *via* water electrolysis using renewable energy (Turner et al., 2008). The advantage of using MOB and HOB is the “barrier” between protein production and waste treatment, as they can utilize gaseous substrates that originate from resource recovery (e.g., biogas, ammonia). This metabolism couples CH_4_ utilization and CO_2_ mitigation with added-value resource recovery, without requiring any further treatment (AlSayed et al., 2018). In addition, ammonia could be electrochemically extracted (Khoshnevisan et al., 2019) or stripped from the digestate, therefore providing a “clean” nitrogen source for MP production. The extracted ammonia could additionally serve as a means for pH correction (increase), therefore decreasing the costs for chemicals. Due to these reasons, the use of raw or upgraded biogas for the production of MP has recently attracted attention (Acosta et al., 2019; Khoshnevisan et al., 2019).

To our knowledge, only a few HOB strains, belonging mainly to the genera *Cupriavidus* (previously known as *Hydrogenomonas* or *Alcaligenes*), *Azohydromonas*, *Herbaspirillum*, *Pseudomonas*, *Paracoccus*, *Sulfuricurvum*, *Azonexus*, and *Xanthobacter* have been evaluated for MP production (Foster et al., 1969; Kunasundari et al., 2013; Matassa et al., 2016; Dou et al., 2019; Hu et al., 2020; Alvarado et al., 2021). At the same time, knowledge about autotrophic HOB that can be used to produce MP remains limited, while information on the use of MOB for MP production is restricted to *Methylococcus capsulatus* (Bath). While the combination of MOB and HOB has been demonstrated using enriched mixed cultures (Acosta et al., 2019), there are no reports available on the potential benefits of combining specific strains of MOB and HOB.

In this work, we investigated the co-cultivation of MOB and HOB to identify the best combination for the production of MP (Figure 1). An isolation campaign was set to obtain a diverse group of novel HOB, capable of autotrophic metabolism. A set of MOB strains, that were obtained from a culture collection, were co-cultivated with selected HOB isolates and the best combinations based on the cell density were identified, hypothesizing that the co-culture will perform better towards MP production than the individual pure cultures. The ten best combinations were grown in serum vials to identify the best co-cultures in terms of nutritional characteristics, considering the abundance of each species, total protein content, amino acid composition and improvements compared to the individual pure cultures.

## Materials and Methods

**FIGURE 2:**
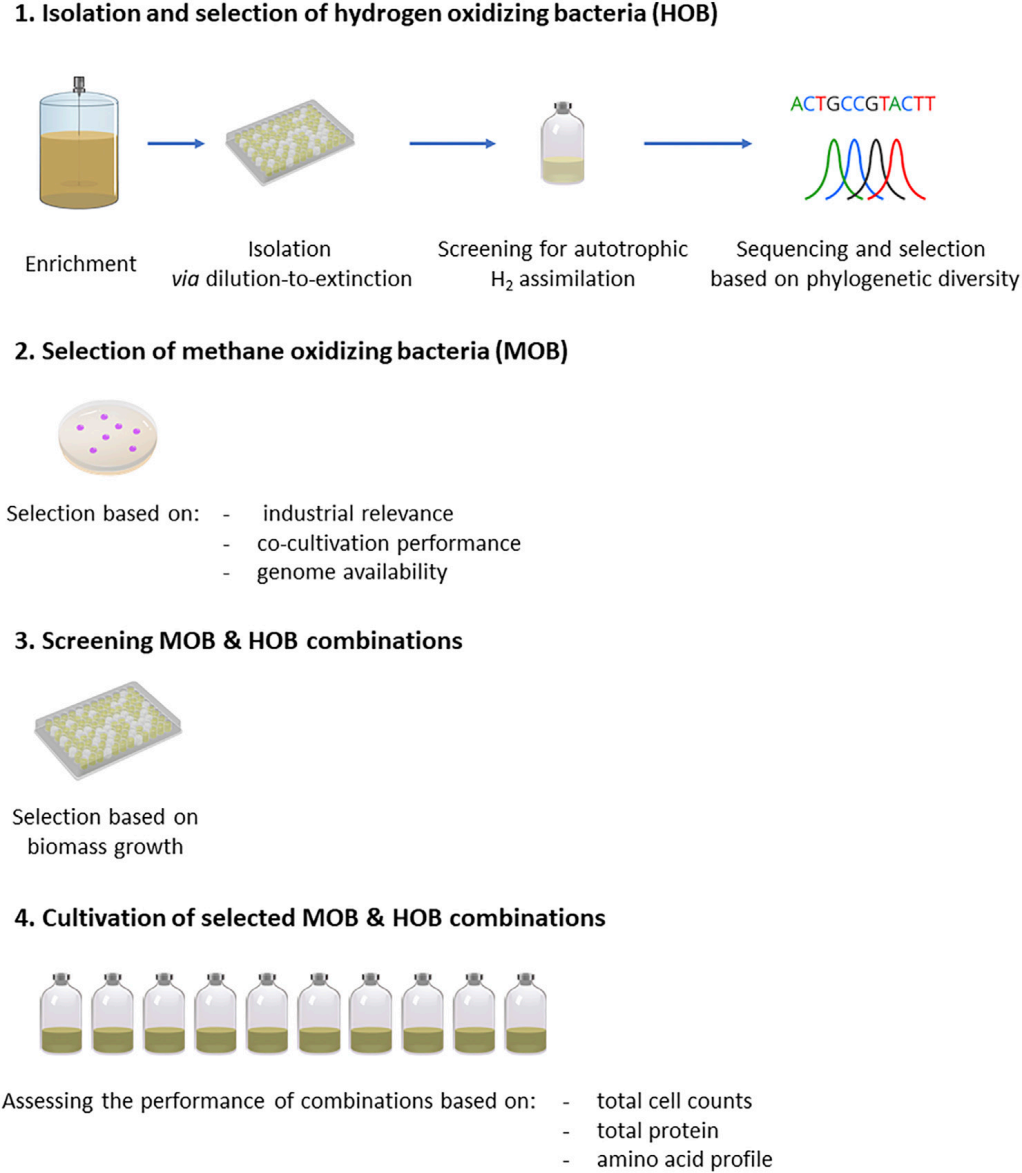
Experimental methodology followed for the enrichment, selection, screening and assessment of pure cultures of HOB and MOB as well as their combination, in the context of microbial protein production.

In order to evaluate MP production by co-cultivation of HOB and MOB, first HOB enrichments were performed (*Enrichment of Hydrogen Oxidizing Bacteria*), followed by isolation of new HOB strains (*Isolation and Selection of Hydrogen Oxidizing Bacteri*a). Next, relevant MOB were selected (*Selection and Growth Conditions of Methanotrophic Strains*) and were co-cultivated in 96 well plates with the HOB to screen the promising combinations (*Screening of Co-cultures of Isolated and Selected Strains in Microtiter Plates*). The best performing combinations in terms of growth were cultivated in serum vials to identify potential benefits from the co-cultivation of HOB and MOB and assess their nutritional properties (*Cultivation of Selected Co-cultures in Serum Vials*). The experimental methodology is illustrated in Figure 2.

### Isolation, Selection, and Cultivation of Strains

#### Enrichment of Hydrogen Oxidizing Bacteria

To obtain diverse inocula, rich in HOB, enrichments were performed as described in Supplementary Material, *Enrichment of Hydrogen Oxidizing Bacteria*. In brief, soil samples were initially enriched under a sequential batch regime, using medium for chemolithotrophic growth (DSMZ medium 81, Supplementary Table S1) while H_2_ and O_2_ were produced through water electrolysis. After 10 cycles of batch enrichment, the operational mode was changed to continuous, using four 1 L glass reactors with a working volume of 0.4 L, operated at a hydraulic retention time (HRT) of 120 h (Ehsani, 2020). Next, four opaque serum vials with a final volume of 120 ml containing mineral medium were inoculated with the enriched culture to result in 20 ml final volume, with a headspace composition of 2% O_2_, 10% H_2_, 10% CO_2_, and 78% N_2_. After an intensive enrichment period of 3 months (Supplementary Material, *Enrichment of Hydrogen Oxidizing Bacteria*) a sample was taken for the isolation campaign.

#### Isolation and Selection of Hydrogen Oxidizing Bacteria

After the enrichment, an isolation campaign was set to obtain novel HOB strains. Two inocula were used: the enrichment originating from the soil sample (*Enrichment of Hydrogen Oxidizing Bacteria*) and one sample obtained from an autotrophic biocathode (Prévoteau et al., 2019). The two different samples were diluted to extinction (DTE) in two different 96-well plates as described in Hoefman et al. (2012). Briefly, the cultures were diluted 10 to 10^10^ times in medium for chemolithotrophic growth, in microtiter plates at a final working volume of 0.25 ml. The plates were then placed in an airtight jar, and were incubated under 23% H_2_ (Air Liquide, Liège, Belgium), 39% N_2_, 19% O_2_ and 19% CO_2_ (Linde Gas Benelux BV, Schiedam, Netherlands), at 28°C for 3 days. Next, the optical density (OD) at 600 nm was measured using an Infinite® M200 Pro microplate reader (Tecan™, Männdorf, Switzerland) (Supplementary Material, *Optical Density*), and the wells with the highest dilution that presented growth were selected for plating. These were plated on 1, 10, and 100% trypticase soy agar (TSA) and on DSMZ medium 81 plates, at 28°C. The TSA plates were incubated under atmospheric air to verify the ability of the isolated strains to grow heterotrophically, while the DSMZ 81 plates were incubated in a jar containing 23% H_2_, 39% N_2_, 19% O_2_, and 19% CO_2_ to verify their ability to grow autotrophically, using H_2_ as electron donor. After 5 days, single colonies were transferred in liquid DSMZ 81 in serum vials containing H_2_, N_2_, O_2_, and CO_2_ as described above. The H_2_ concentration in the headspace was monitored, to verify the H_2_ utilization by the HOB. The cultures that failed to consume the H_2_ contained in the headspace were removed from the study, while the cultures that showed hydrogen oxidizing activity were retained. This process yielded 42 isolates.

#### Selection and Growth Conditions of Methanotrophic Strains

Nine methane oxidizing bacteria (MOB) were obtained from the Belgian Coordinated Collection of Microorganisms (BCCM-LMG; Ghent, Belgium) (Table 1). The selection criteria were their industrial relevance (e.g., *Methylococcus capsulatus*), previous data on the co-cultivation performance of *Methylosinus* spp. and *M. methanica* (Kerckhof et al., 2016) and genome availability (except for MOB 6). Unless in co-cultivation or when stated otherwise, the MOB were grown on nitrate mineral salts medium (NMS (Whittenbury and Wilkinson, 1970), Supplementary Table S2) at 28°C under a 50% CH_4_ atmosphere (on semisolid media) or a 20% CH_4_ atmosphere (liquid media, shaking at 120 rpm). To assess the purity of these obligate methanotrophs, plating on 10% trypticase soy agar (TSA) was performed at 28°C for up to 10 days to assess that no colonies were formed, as well as regular full-length 16S rRNA gene sequencing (Supplementary Material, *DNA Extraction* and *Sanger Sequencing*).

**TABLE 1:** Methanotrophic bacteria used in the present study, with collection number and genome accession (if available).

Species	Strain	Assigned name	Genome (IMG status)
***Methylomonas koyamae***	LMG 26261 (Hoefman R-45378)	MOB1	PRJNA315276 (Permanent draft)
***Methylosinus sp.***	LMG 26262 (Hoefman R-45379)	MOB2	PRJNA315280 (Permanent draft)
***Methylomonas methanica***	LMG 26612 (Hoefman R-45363)	MOB3	PRJNA315271 (Permanent draft)
***Methylomonas methanica***	LMG 26614 (Hoefman R-45371)	MOB4	PRJNA315272 (Permanent draft)
***Methylococcus capsulatus***	LMG 26900	MOB5	PRJNA21 (Finished)
***Methyloparacoccus murrelli***	LMG 27482 (Hoefman R-49797)	MOB6	Not available
***Methylomonas koyamae***	LMG 27769 (Hoefman R-49807)	MOB7	PRJNA315278 (Permanent draft)
***Methylocystis hirsuta***	LMG 27832 (Hoefman R-43155)	MOB8	PRJNA487728 (Not in IMG or GOLD)
***Methylovulum psychotolerans***	LMG 29227	MOB9	PRJNA418066 (Permanent draft)

#### Screening of Co-Cultures of Isolated and Selected Strains in Microtiter Plates

The 9 MOB and 19 HOB isolates that were selected from the isolation campaign (based upon phylogenetic diversity, *Isolation Campaign*) were combined (one MOB with one HOB) to obtain the best performing combinations in terms of growth. The experiments were performed in 96-well plates, at a final working volume of 0.2 ml. The MOB and HOB pure cultures were diluted in liquid mineral medium, at a final cell density of 10^6^ cells/mL. The medium used during all co-cultivations was DSMZ medium 81 (Supplementary Table S1) supplemented with the NMS trace-elements solution with abundant Cu to allow particulate methane mono-oxygenase to be expressed (Semrau et al., 2018). Next, a volume of 0.2 ml was transferred in the respective well for the growth of the individual strains, while for the combinations of one HOB and one MOB, a volume of 0.1 ml and from each strain was used (i.e., to achieve a final volume of 0.2 ml). The plates were placed in a gas-tight jar and flushed with 80:20 v/v H_2_/CO_2_ mixture. Next, CH_4_ and O_2_ were added to the headspace to achieve a final headspace composition of 16.5% O_2_, 29% H_2_, 12.5% CO_2_, and 42% CH_4_. The initial OD was measured at day 0, and subsequently at days 3, 6, and 10. After OD measurements, the 96-well plates were placed again in the gas-tight jar, and the gases were replenished as described. The best combinations with respect to joint biomass growth were determined through flow cytometry and selective plate counts, in biological duplicates.

#### Cultivation of Selected Co-Cultures in Serum Vials

The eight best combinations were selected based on the plating of the combinations of the 9 MOB with the 19 HOB. Additionally, *Cupriavidus necator* LMG 1201 (CNEC) and *Xanthobacter autotrophicus* R-75741 (XAUT) were combined with the best growing MOB, *Methyloparacoccus murrelli* LMG 27482, as established by the results of plating and flow cytometry measurements. *C. necator* is one of the most well-described species for hydrogen-driven MP production (Yu, 2018), whereas we recently identified *X. autotrophicus* as an autotrophic, nitrogen-fixing HOB (Hu et al., 2020). The MOB and HOB pure cultures and their combinations (inoculated at equal initial cell counts of MOB and HOB) were inoculated in DSMZ medium 81 containing trace elements, at a final cell density of 10^6^ cells/mL and a final volume of 20 ml and *ca.* 100 ml of headspace, using opaque serum vials. All bottles were flushed with a mixture of H_2_/CO_2_ (80/20% v/v) and O_2_ and CH_4_ were subsequently supplemented, with the initial concentrations in the gas phase being of 46% H_2_, 12% CO_2_, 30% O_2_, and 12% CH_4_ (Supplementary Material, *Selection of Gas Composition*). The cultures were incubated at 28°C under a rotary shaking of 120 rpm. All cultures were grown in biological triplicates. The cell counts through flow cytometry, total protein and amino acid analysis were performed after five days of growth.

### Individual Quantification

#### Selective Plate Counts

To count the number of individual HOB or MOB in the co-cultures, a selective plate counting approach was utilized, by counting the number of colony forming units (CFU) on NMS plates, cultivated at 28°C under 50% CH_4_ atmosphere (exclusively counting the MOB, which are all obligate methanotrophs (Whittenbury et al., 1970)) and 100% TSA (exclusively counting the HOB, which are mixotrophs). Colonies were counted at variable time intervals between one and 10 days, to account for the variation in the growth rates between the HOB isolates. To reduce counting bias, plates were scanned using an Epson Perfection V800 Photo scanner (Seiko Epson Corporation, Suwa, Nagano, Japan) with the VueScan software (v.9.5.57, Hamrick Software, Sunny Isles Beach, Florida, United States). High-resolution scans were subsequently processed using the open source Fiji software (v. 2.0.0 (Schindelin et al., 2012)) by isolating only the plate in the image, subtracting the background with a rolling ball radius set to 50 through 100 pixels (depending upon image contrast), and making the image monochrome (binary) to compensate for small aberrations in reflection. A Gaussian blur filter was applied to make the colonies rounder, and either outliers in background noise were removed, or thresholding and contrast/brightness adjustments were employed. If the colonies were overlapping at the edges, a watershed separation was used. Ultimately, colony counting was performed using the “Analyze Particles” function in Fiji. The size was set in pixel units and was dependent upon the size of the cells and remaining background noise. Circularity was set between 0.4 (when colonies were not very circular) and 1; and masks were inspected to verify if counts reflected colonies on the plate.

#### Flow Cytometry

To assess initial inoculum concentrations (10^6^ cells/mL) and not miss cells that were potentially rendered into a viable but not culturable state in co-cultivation, microbial flow cytometry was performed to determine the total cell concentrations, as described before (Van Nevel et al., 2017). In brief, samples were measured on a Becton-Dickinson FACSVerse 3-laser (405/488/640 nm) cytometer (BD, Franklin Lakes, New Jersey, United States), after dilution in phosphate buffered saline (PBS; Thermo Fischer Scientific, Belgium) and staining with 1x SYBR Green I (Life Technologies) for 20 min at 37°C. The FACSVerse instrument used was equipped with a volumetric sensor to allow for absolute quantification without the need for counting beads. In measurements where the volumetric sensors failed, the volumes were imputed based upon robust linear models from the “rlm” function in the R package MASS (Venables and Ripley, 2002) (Supplementary Table S7, S8). The excitation of the SYBR green 1 was performed by the 488 nm laser and detected in the FITC-A signal (bi-exponential scaling was used for visualization in the FACSSuite software) with a bandpass filter of 527/32 (following a 507 LP). This channel was also used as a trigger threshold. For microbial fingerprinting (*Data Analysis*), we used channels FSC-A, SSC-A, FITC-A (527/32) and PerCP-Cy5.5-A (700/54 after 665LP).

### Total Protein and Amino Acids

The total protein analysis was performed using the DC™ Protein Assay (Bio-Rad Laboratories Inc., Hercules, United States) in triplicate for each sample (Supplementary Material, *Total Protein Quantification*). The essential amino acid (EAA) and conditionally EAA (CEAA) content was determined as described in Muys et al. (2019). Briefly, pelletized biomass was subjected to acid hydrolysis (6M HCl for 24 h at 110°C) in the absence of oxygen. Next, the hydrolysate was derivatized using the Phenomenex EZ:faast amino acid protocol, with norvaline as internal standard, while bovine serum albumin (BSA) was used as a control to determine amino acid recovery after acid hydrolysis. Finally, the quantification of the amino acids was performed using gas chromatography equipped with mass spectrometry. The EAA histidine, lysine, phenylalanine, threonine, valine, leucine and isoleucine; and CEAA glutamine, glycine, proline are presented. Methionine, cysteine, tyrosine, and tryptophan are not included in this work, since they are (partially) destroyed during acid hydrolysis. Therefore, these results are not included in the graphs but are presented in Supplementary Table S3 for comparison.

### Sanger Sequencing

After DNA extraction (Supplementary Material, *DNA extraction*), a near full-length amplicon (27F/1492R) of the 16S rRNA gene of individual isolates was purified, and sent for Sanger sequencing (LGC Genomics Gmbh, Berlin; Supplementary Material, *Sanger Sequencing*). The sequences were classified with the Ribosomal Database Project Naïve Bayesian Classifier (Wang et al., 2007), with the 16S rRNA gene training set 16 at a 80% confidence threshold (rdp.cme.msu.edu), SILVA (Quast et al., 2013) nr release 138 ACT (Pruesse et al., 2012) with default options for classification of SSU rRNA genes (https://www.arb-silva.de/aligner/) and NCBI Blast. The results were compared, and the most appropriate classification was assigned to the samples.

### Data Analysis

All data was analyzed using the R language for statistical programming, version 4.0.4. When performing hypothesis testing for pairwise comparisons, a (pairwise) Wilcoxon Rank Sum test with Holm’s method to adjust the *p*-values was employed. Significance was tested at the *α*-level of 5%. When normality (and homoscedasticity) assumptions were testable and met, a (Welch) *t*-test was used.

For the analysis of the flow cytometry data, we employed the methods as described by Rubbens et al. (2017) to distinguish the individual HOB and MOB concentrations in the mixture, leveraging the implementations in the Phenoflow package of Props et al. (2016). In brief, we trained a random forest classifier to all potential pairings of the axenic cultivations in modified DSMZ 81 medium (*Cultivation of Selected Co-Cultures in Serum Vials*), and applied this classifier to the co-cultivations at the time of sampling for protein analyses. To assure data quality, we employed “flow_auto_qc” as implemented in flowAI (Monaco et al., 2016), on every combination with at least 1,000 cells. Samples with less than 1,000 cells recorded were not taken along for downstream analysis, due to their limited value for predictions. Analysis code has been made available on github.com/CMET-UGent/Kerckhof_Sakarika_2021.

## Results

### Isolation Campaign

To obtain a diverse set of autotrophic HOB, a total of 42 isolates were obtained through dilution plating and dilution to extinction (Supplementary Figure S1). Based on the length of the sequences obtained by Sanger sequencing and the phylogenetic diversity of the isolates, 19 strains were selected for further screening (Table 2).

**TABLE 2:** Properties of selected HOB isolates for preliminary screening.

Classification	Assigned name	Source	BCCM/LMG accession number	Genbank identifier 16S rRNA
***Hydrocarboniphaga effusa***	HOB 1	Biocathode	LMG 32163	MT625930
***Ancylobacter*** ** sp.**	HOB 2	Enriched soil samples	LMG 31925	MT625931
***Bacillus*** ** sp.**	HOB 3	Enriched soil samples	LMG 31926	MT625932
***Pseudacidovorax intermedius***	HOB 4	Enriched soil samples	LMG 31927	MT625933
***Achromobacter*** ** sp.**	HOB 5	Enriched soil samples	LMG 31928	MT625934
***Acinetobacter venetianus***	HOB 6	Biocathode	*	MT625935
***Hydrogenophaga electricum***	HOB 7	Biocathode	LMG 32162	MT625936
***Azonexus*** ** sp.**	HOB 8	Enriched soil samples	*	MT625937
***Rhodococcus*** ** sp.**	HOB 9	Enriched soil samples	LMG 31929	MT625938
***Xanthobacter*** ** sp.**	HOB 10	Enriched soil samples	LMG 32161	MT625939
***Pseudomonas*** ** sp.**	HOB 11	Enriched soil samples	LMG 31930	MT625940
***Dermacoccus*** ** sp.**	HOB 12	Enriched soil samples	*	MT625941
***Paenibacillus*** ** sp.**	HOB 13	Enriched soil samples	LMG 32160	MW287568
***Xanthobacteraceae*** ** sp.**	HOB 14	Enriched soil samples	*	MT625943
***Pseudomonas*** ** sp.**	HOB 15	Enriched soil samples	LMG 31931	MT625944
***Pseudomonas*** ** sp.**	HOB 16	Enriched soil samples	LMG 32159	MZ701924
***Pseudacidovorax*** ** sp.**	HOB 17	Enriched soil samples	*	MW287570
***Pseudomonas*** ** sp.**	HOB 18	Enriched soil samples	LMG 31932	MT625947
***Paenibacillus*** ** sp.**	HOB 19	Enriched soil samples	*	MT625948

*Strain was lost before submission to culture collection was possible–recovery attempts failed.

### Preliminary Screening

**FIGURE 3:**
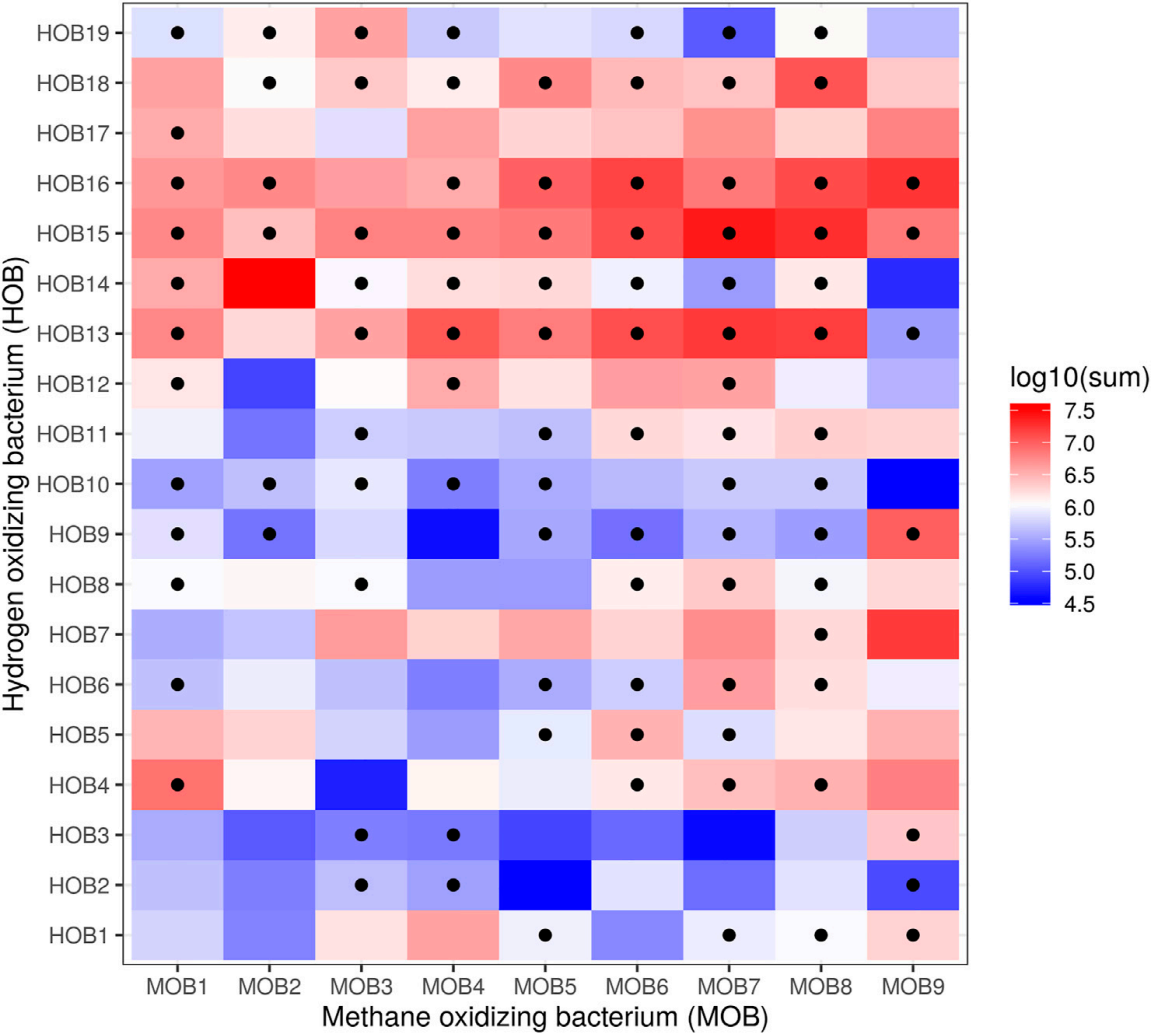
Heatmap of log_10_ CFU/mL plate counts of combinations of autotrophic hydrogen oxidizers (counted on DSMZ medium 81) and methanotrophs (counted on NMS), after a 10^4^ dilution in PBS. Dots show combinations with a ratio of HOB/MOB between 0.5 and 2. The combinations of MOB with CNEC and XAUT were not evaluated through selective plate counting.

To select the best potential combinations of HOB and MOB, we performed a co-cultivation assay in microtiter plate format of all 171 combinations (Figure 3) and evaluated the composition using selective plating as described in *Individual Quantification*. From those combinations, 10 were selected based upon the ability of the HOB and MOB to grow together and the best growing MOB (i.e., MOB6) was combined with *C. necator* and *X. autotrophicus* for reference purposes (results of selective plating; Table 3). The results of plating were supported by inference from the flow cytometry fingerprints (Supplementary Figures S2–4), and every co-culture had a higher cell concentration than could be expected from a 1:1 combination of the cell concentrations of the axenic cultures taken at the same time-point in the cultivation (Supplementary Table S3).

**TABLE 3:** Ratio of HOB to MOB and measured CFU concentration after 4 days of incubation in a microtiter plate. Ratios were inferred from selective plating. Values of ratios are expressed as mean values ± standard deviation (*n* = 3).

Combination	Ratio HOB to MOB	Measured CFU concentration (CFU/ml)
**MOB1·HOB7**	0.60^a^	1.6 × 107^a^
**MOB4·HOB13**	1.16 ± 0.01	1.19 × 107 ± 0.7 × 10^6^
**MOB5·HOB16**	1.56 ± 0.42	1.26 × 107 ± 2.8 × 10^6^
**MOB6·HOB15**	1.30 ± 0.55	1.20 × 107 ± 0.3 × 10^6^
**MOB6·HOB16**	0.83 ± 0.12	1.13 × 107 ± 3.3 × 10^6^
**MOB8·HOB13**	0.93 ± 0.32	1.78 × 107 ± 2.0 × 10^6^
**MOB8·HOB15**	0.89 ± 0.01	1.56 × 107 ± 3.4 × 10^6^
**MOB8·HOB18**	0.76 ± 0.09	1.04 × 107 ± 1.3 × 10^6^

a No standard deviation could be calculated because there was only one observation.

### Selected Combinations

#### Co-cultivation Dynamics

To assess the biomass production and composition, we employed flow cytometric fingerprinting and machine learning (Supplementary Figure S5). The total cell concentration was measured for each selected combination as well as its axenic constituents. The axenic growth was used to estimate how much growth could be expected in a 1:1 combination of both strains. Six out of ten combinations have a significantly higher cell density than expected based on the axenic cultures’ cell concentration (Table 4). Only the combination of MOB8 with HOB18 exhibited a lower cell density in co-culture than could be expected from the axenic growth (not significant at the 5% confidence level, *p* = 0.482, 2-sided alternative). Based upon the random forest model predictions, all combinations exhibited co-culture compositions at day 5, where the HOB cell concentration was between 30 and 85% of the MOB cell concentration, except for combination MOB6·HOB16, where the amount of MOB and HOB was comparable; and the combination MOB6·CNEC where 3.59 ± 2.18 (*n* = 3) times more HOB cells (i.e., CNEC) were present than MOB (Table 5).

**TABLE 4:** Expected and observed cell densities of the combinations and their ratio.

Combination	Ratio	Final cell concentration (cells/mL)	Expected cell concentration with equal mixing ratios (cells/mL)
**MOB1·HOB7**	1.37^a^	1.36 × 10^7^ ± 2.1 × 10^6^	0.99 × 10^7^ ± 0.90 × 10^7^
**MOB4·HOB13**	1.11	2.13 × 10^7^ ± 2.3 × 10^6^	1.39 × 10^7^ ± 1.79 × 10^7^
**MOB5·HOB16**	1.14^a^	4.50 × 10^7^ ± 3.5 × 10^6^	3.96 × 10^7^ ± 3.20 × 10^7^
**MOB6·HOB15**	1.18^a^	5.25 × 10^7^ ± 5.7 × 10^6^	4.44 × 10^7^ ± 3.86 × 10^7^
**MOB6·HOB16**	1.43^a^	5.30 × 10^7^ ± 4.0 × 10^6^	3.71 × 10^7^ ± 3.15 × 10^7^
**MOB8·HOB13**	1.46^a^	1.38 × 10^7^ ± 2.3 × 10^6^	0.94 × 10^7^ ± 0.81 × 10^7^
**MOB8·HOB15**	1.13^a^	5.35 × 10^7^ ± 5.5 × 10^6^	4.75 × 10^7^ ± 4.03 × 10^7^
**MOB8·HOB18**	0.67	1.98 × 10^7^ ± 4.8 × 10^6^	2.95 × 10^7^ ± 2.30 × 10^7^
**MOB6·XAUT**	1.52	5.57 × 10^7^ ± 3.8 × 10^6^	3.65 × 10^7^ ± 3.09 × 10^7^
**MOB6·CNEC**	3.54	23.1 × 10^7^ ± 10.0 × 10^7^	6.54 × 10^7^ ± 5.94 × 10^7^

a Indicates where a significant difference in densities could be observed at the 5% significance level (2-sided alternative). Values are reported as averages ±standard deviation (*n* = 3). XAUT, Xanthobacter autotrophicus R-75741; CNEC, Cupriavidus necator LMG 1201

**TABLE 5:** Ratio of HOB to MOB cell densities at the time of sampling. Values are reported as averages ± standard deviation (*n* = 3). XAUT, *Xanthobacter autotrophicus* R-75741; CNEC, *Cupriavidus necator* LMG 1201.

Combination	Ratio HOB cell concentration to MOB cell concentration
**MOB1·HOB7**	0.34 ± 0.04
**MOB4·HOB13**	0.40 ± 0.09
**MOB5·HOB16**	0.85 ± 0.10
**MOB6·HOB15**	0.28 ± 0.04
**MOB6·HOB16**	1.01 ± 0.05
**MOB8·HOB13**	0.31 ± 0.05
**MOB8·HOB15**	0.30 ± 0.03
**MOB8·HOB18**	0.71 ± 0.20
**MOB6·XAUT**	0.58 ± 0.20
**MOB6·CNEC**	3.59 ± 2.18

#### Total Protein and Amino Acids

The total protein content of the (co-)cultures was quantified in all samples after 5 days of growth (Figure 4.1; Supplementary Table S4). The highest protein content (0.578 pg/cell) for the MOB cultures was obtained by MOB1, while the lowest (0.0825 pg/cell) was obtained by MOB4. The pure HOB cultures presented a less broad protein content range (0.0303–0.362 pg/cell) than the MOB, with HOB13 presenting the highest protein content (0.362 pg/cell). One out of the ten combinations, MOB8·HOB18, presented a 1.38 times higher protein content than the one estimated based on the axenic cultures while MOB1·HOB7 presented a similar protein content (1.02 times higher) than the individual MOB and HOB strains.

**FIGURE 4:**
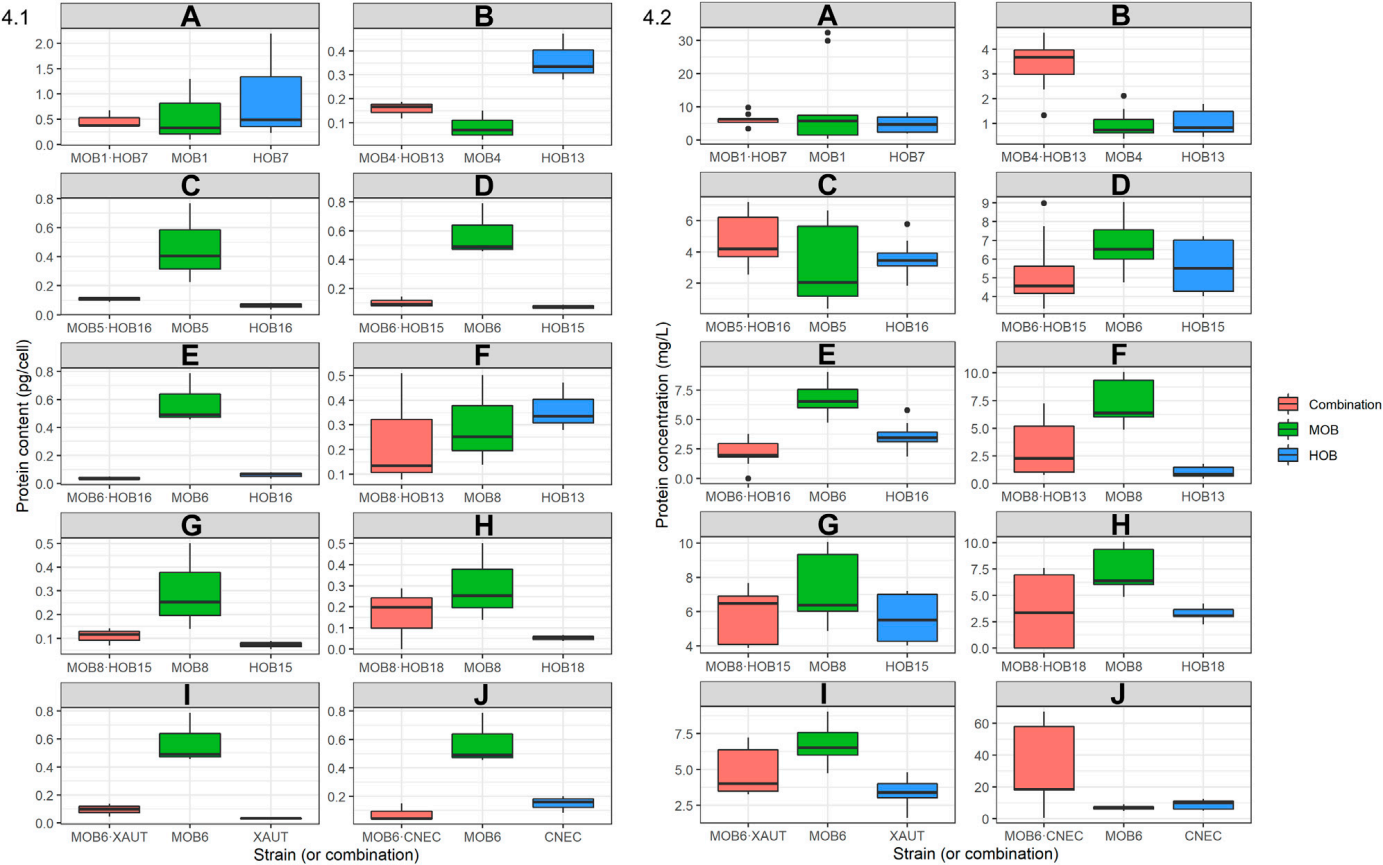
Box-and-whisker plots of 4.1 protein content (pg/cell) and 4.2 protein concentration (mg/L) of the individual HOB and MOB cultures as well as selected combinations as determined after 5 days of growth. The protein concentration was quantified using the BioRad DC protein assay with bovine serum albumin (BSA) as standard. Outliers are shown with black dots outside of the box-and-whisker plots, the median is shown as a horizontal black bar inside of the plots. Axes between subplots are not constant. The complete dataset is presented in Supplementary Tables S4, S5.

The effect of the co-cultivation was more prominently reflected in the protein concentration (Figure 3.2; Supplementary Table S5). Five out of the ten combinations exhibited higher protein concentration than expected based upon an equal mixture of the axenic cultures. These combinations, in declining order, were MOB6·CNEC (3.79 times higher)> MOB4·HOB13 (1.65 times higher)> MOB1·HOB7 (1.17 times higher)> MOB8·HOB15 (1.09 times higher)> MOB8·HOB13 (1.06 times higher). These combinations, except for the latter, resulted in statistically significant higher protein concentration than expected based upon equal mixtures of the axenic cultures (Supplementary Table S5). The other five combinations resulted in significantly lower protein concentration than expected, with MOB6·HOB16 resulting in 2.7 times lower protein. Regardless of the comparison with the expected values, the highest protein concentration was achieved by MOB6·CNEC (29.4 mg/L) and the lowest was noted by MOB6·HOB16 (2.14 mg/L).

**FIGURE 5:**
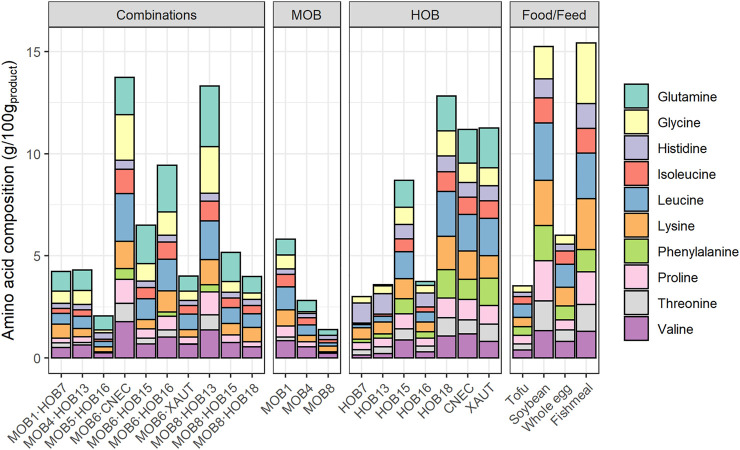
Composition of essential and conditionally essential amino acids in the biomass of the combined methane oxidizing bacteria (MOB) and hydrogen oxidizing bacteria (HOB) cultures as well as individual MOB and HOB strains. The amino acid content is presented in g/100g_product_, taking into account 5% of moisture (Chemical Composition 1-Unibio, 2021). The amino acid composition of common food and feed ingredients are presented for comparison (in g/100g_product_). Essential amino acids: histidine, lysine, phenylalanine, threonine, valine, leucine, isoleucine; Conditionally essential amino acids: glutamine, glycine, proline. Methionine, cysteine, tyrosine, and tryptophan are not included in this graph, since they are (partially) destroyed during acid hydrolysis. MOB 5 and MOB 6 were not included due to poor data quality. The glutamine content of the food and feed ingredients was not reported in the respective references. The full dataset can be found in the Supplementary Tables S6, S7.

Overall, the highest EAA and CEAA content was exhibited by MOB6·CNEC (14.5 g/100 g_product_) and MOB8·HOB13 (14.0 g/100 g_product_), followed by HOB18 (13.5 g/100 g_product_) (Figure 5). The EAA and CEAA content of the combinations ranged between 2.16 and 14.5 g/100 g_product_, for the HOB between 3.16 and 13.5 g/100 g_product_, while the EAA and CEAA content of MOB remained at lower levels (0.37–6.12 g/100 g_product_). The highest EAA and CEAA contents for the HOB were noted by HOB18 (13.5 g/100 g_product_)>XAUT (11.8 g/100 g_product_)>CNEC (11.8 g/100 g_product_), while the MOB with the highest EAA and CEAA content were MOB1 (6.12 g/100 g_product_)>MOB4 (2.96 g/100 g_product_)>MOB8 (1.47 g/100 g_product_). Five out of the ten combinations resulted in higher EAA and CEAA content than expected based upon the pure cultures (1.4–6.1 times higher; Figure 5). MOB8·HOB13 presented 6.1 times higher EAA and CEAA content compared to the pure cultures, followed by MOB6·HOB16 (4.6 times higher content) and MOB6·CNEC (2.4 times higher content). Five out of the ten combinations presented similar EAA and CEAA content than estimated (0.96–1.44 times), while the rest of the cultures presented lower content than estimated (0.56–0.69 times; Supplementary Table S8).

The co-cultivation also affected the amino acid distribution, compared to the pure cultures, as can be seen by the distribution of the branched-chain amino acids (BCAA) leucine, isoleucine, and valine (Supplementary Figure S6). In all cases, HOB presented the lowest valine content, which increased (1.2–2.9 times) when they were combined with MOB, where the combinations MOB4·HOB13 and MOB1·HOB7 presented the highest increase (2.9 times). Similarly, 9 out of the 10 combinations had higher isoleucine content than the respective HOB, with MOB1·HOB7 and MOB8·HOB13 presenting the highest increase (3.3 and 3.1 times higher, respectively). The leucine content was higher for 7 out of the 10 combinations compared to the content of the respective HOB, with MOB1·HOB7 and MOB8·HOB13 presenting the highest increase (2.9 and 2.5 times higher, respectively).

## Discussion

### Co-Cultivation of MOB and HOB for Microbial Protein Production Could Enable System Stability and Higher Resource Utilization Efficiency

The general advantages of using HOB for MP production were summarized from Dou et al. (2019): 1) compared to other MP types, HOB have higher protein content (i.e., 40-60% for microalgae, 30–45% for fungi and 45–55% for yeasts) (Nasseri et al., 2011); 2) they are metabolically versatile, and can switch from autotrophic to heterotrophic mode; 3) even though they are autotrophic, they do not get limited by light availability; 4) they contain intracellular products with prebiotic functions (*i.e.,* polyhydroxybutyrate (PHB)); and 5) they fix CO_2_ to protein. Similar advantages can be reported for MOB. In addition, 4) instead of using costly mineral media, low-strength wastewaters can alternatively be used for the cultivation of MOB and HOB. As an example, *Methylococcus capsulatus* was able to grow on potato wastewater, with the only external supply being methane (Rasouli et al., 2018). In this manuscript, we describe the use of co-cultivation of MOB and (newly isolated) autotrophic HOB for the production of MP. While the general advantages for MP production with either of these organisms are summarized above, their co-cultivation could offer distinct advantages from a metabolic viewpoint, in terms of carbon utilization efficiency and recovery and, potentially, with respect to system stability.

It is known that MOB benefit from co-cultivation in terms of oxidation rates and the consumption of byproducts of methane oxidation (Schmaljohann, 1991; van der Ha et al., 2012; Beck et al., 2013; Stock et al., 2013; Ho et al., 2014; Iguchi et al., 2015; Oshkin et al., 2015; Kerckhof et al., 2016). Given their capability of mixotrophic growth, HOB can serve as ideal co-culture partners to provide this functionality to the MOB. HOB can remove the organic carbon that is “leaked” by the MOB, reducing available niches for potential contaminants of the MP production process, as documented for *Methylococcus capsulatus* (Bath) in co-culture with the heterotrophic *Ralstonia* spp., *Brevibacillus* and *Aneurinibacillus* spp. (Bothe et al., 2002). Other examples of process stabilization by co-cultures with MOB have been documented for groundwater pollutant degradation (Hršak and Begonja, 1998), denitrification (Modin et al., 2007) and production of biopolymers (such as PHB) from CH_4_ (Karthikeyan et al., 2015). This metabolic coupling could also be relevant when the process becomes oxygen-limited, which could trigger methane biocatalysis with extracellular metabolites formed, such as formate, acetate, succinate, lactate, 3-hydroxybutyrate and even H_2_ (Kalyuzhnaya et al., 2013), that can be easily consumed by the HOB. The heterotrophic volumetric productivity of HOB is higher than that of autotrophic production (Dou et al., 2019), which can be attributed to gas-liquid mass transfer limitations. Therefore, the addition of HOB to MOB cultures has a benefit that, apart from eliminating the soluble metabolites of methanotrophic bacteria (which can further cause inhibition), it increases biomass concentration and productivity. Finally, it has been estimated that the autotrophic cultivation of HOB requires roughly 30 times less energy than the cultivation of microalgae (Repaske, 1966), which makes them conceptually more interesting partners for MOB than microalgae.

Even though co-cultivation with heterotrophic organisms results in enhanced performance of methanotrophs, they are not able to utilize CO_2_. The combination of MOB with microalgae enables CO_2_ utilization (van der Ha et al., 2012) however, the need for a light source and the low growth rate of microalgae would increase the production costs (growth rates of 0.00913–0.0320 h^−1^ for photoautotrophic microalgae (Kim et al., 2013); 0.420 h^−1^ for HOB (Ishizaki and Tanaka, 1990) and 0.0430 h^−1^ for MOB (Yazdian and Hajizadeh, 2005)). Under autotrophic growth, the HOB will consume CO_2_ (both from biogas and produced from methane oxidation) and, hence, decrease the potential for medium acidification. From the perspective of HOB, MOB are primary producers of carbon both for autotrophic growth (CO_2_) as well as mixotrophic growth (discussed above). This contributes to an enhanced carbon recovery into MP as both the CH_4_-derived CO_2_ as well as the CO_2_ already present in the biogas can be assimilated by the HOB. Due to their potential for mixotrophic growth modes, HOB do not require any additional carbon source than biogas (thanks to the MOB’s conversion of CH_4_ into CO_2_ and biomass), and they produce a very limited variety of extracellular metabolites, as the main byproduct of HOB metabolism is water (Volova and Barashkov, 2010). While there have been reports of autotrophic methanotrophic bacteria that can use H_2_, there is no evidence for this metabolism in the liquid culture of proteobacterial MOB (Mohammadi et al., 2019). Where available, we have screened the (finished and draft) genomes of our selected MOB (Table 1) for the presence of RuBisCo and genes of the Calvin-Benson-Bassham (CBB) pathway (which can be an indication of the ability to assimilate CO_2_). Only *M. capsulatus* was found to have RuBisCo, as is consistent with literature (Baxter et al., 2002). The genes necessary for a functional CBB pathway were never all detected in any genome.

From an applied standpoint, to enable the co-cultivation of MOB and HOB with full valorization of the CH_4_ contained in biogas and the H_2_ produced by water electrolysis, air needs to be supplemented (Supplementary Material *Process Integration is Key for Near-Complete Resource Valorization Leading to Lower Environmental Footprint*). Air supplementation would enable 14% increased biomass yield (0.35 kg_biomass_/kg_CODadded_) compared to the use of only O_2_ from water electrolysis (0.31 kg_biomass_/kg_CODadded_). In the case of air addition, the off-gas will contain CO_2_ and N_2,_ at a ratio of 4.9 mol N_2_ per mol CO_2_. In this case, including N_2_-fixing HOB (Hu et al., 2020) and/or N_2_-fixing MOB (Khmelenina et al., 2018) would add value, since the nitrogen utilization of the overall process would be increased. Nevertheless, further investigations are required to validate this approach, since the lower growth rates of N_2_-fixers could compromise the productivity and the overall economics of the proposed process. The addition of air could also lead to a process with a lower carbon footprint, arising from the higher HOB biomass generation. Specifically, it results in 20% lower CO_2_ emissions (2.4 kg_CO2_/kg_biomass_) compared to the base case (3.0 kg_CO2_/kg_biomass_).

### Co-Cultivation of MOB and HOB has Positive Effects on Their Nutritional Properties

**FIGURE 6:**
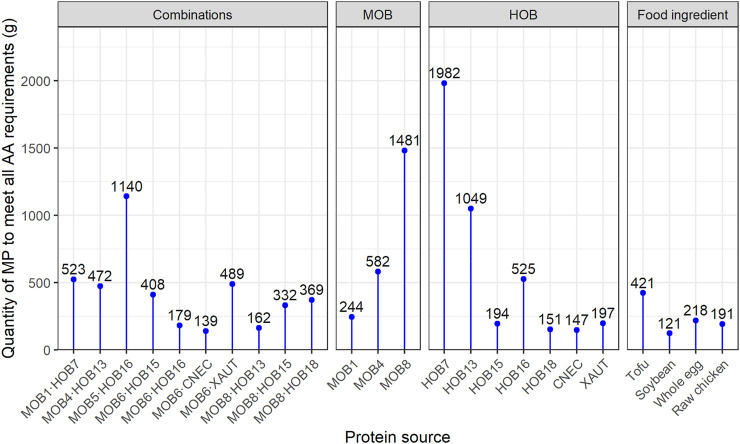
Amount of microbial biomass and various food ingredients needed to cover the daily essential amino acid (EAA) requirements of a 62 kg person (Walpole et al., 2012), as established by WHO/FAO/UNU (2007). The EAA content of microbial protein is calculated taking into account 5% of moisture in the final product (Chemical Composition 1-Unibio, 2021). MOB 5 and MOB 6 were not included due to poor data quality. More information can be found in the Supplementary Tables S9, S10.

Two combinations, namely MOB4·HOB13 and MOB6·CNEC, presented both higher protein concentration and amino acid content than expected based upon the pure cultures, which is an indication of a potentially synergistic relationship between MOB and HOB. The increased protein production during co-cultivation (indicated by the higher protein concentration) is a result of the higher cell density achieved during the co-cultivation. Combining MOB and HOB, instead of growing them as pure cultures, also affected the amino acid profiles, which is evident by the distribution of the BCAA (Supplementary Figure S6). This is noteworthy, since BCAA compose 35% of the EAA in muscle proteins and 40% of the required proteins for mammals (Shimomura et al., 2004). They have a broad range of metabolic and physiologic roles, such as the promotion of protein synthesis and the induction of the immune system (Monirujjaman and Ferdouse, 2014). Overall, the best (co-) cultures in terms of EAA profile were MOB6∙CNEC; CNEC; HOB18; MOB8∙HOB13 and MOB6∙HOB16. The amino acid distribution of these cultures was compared against common food and feed ingredients (Supplementary Figure S6), where it was revealed that MP from MOB and HOB presents a favorable EAA composition. Specifically, the MP produced in the present study had an equal or higher quality of EAA compared to soybean, whole egg and raw chicken, while MP generally contained lower amounts of EAA, mainly histidine, lysine and threonine, compared to tofu and fishmeal (Supplementary Material Comparison of Quality of Microbial Protein from MOB and HOB to Food and Feed Ingredients). The best performing co-culture in terms of amino acid composition and quantity, namely MOB6∙CNEC, requires 27–67% less quantity than chicken, whole egg and tofu, while it only requires 15% more quantity (139 g_ww_) than soybean (121 g_ww_) (Figure 6) to meet the nutritional requirements of an average adult weighing 62 kg (Walpole et al., 2012). In most cases the limiting amino acid was lysine or isoleucine (Supplementary Table S10).

Similar observations were made for other MP types when compared to food/feed ingredients. A bacterial meal produced from natural gas and ammonia, consisting of *Methylococcus capsulatus*, *Alcaligenes acidovorans*, *Bacillus brevis* and *Bacillus firmus* contained higher amounts of threonine and tryptophan compared to fishmeal, while the lysine content was lower (Skrede et al., 1998). Similarly, the HOB *Paracoccus denitrificans* Y5 and *Paracoccus versutus* D6 were found to have higher content in threonine, valine, alanine, and glycine, compared to soybean meal, while they also contained proline which was not present in soybean meal (Dou et al., 2019). When compared to casein, the HOB *Alcaligenes eutrophus* Z1, *Ralstonia eutropha* B5786 and *Seliberia carboxydohydrogena* Z1062 were found to contain higher amounts of threonine, phenylalanine, tryptophan, tyrosine and histidine (Volova and Barashkov, 2010). The amino acid profile of a methanotroph-enriched culture dominated by *Methylococcales* and *Methylophilales* was composed mainly (50%) of aspartic acid, glutamic acid, glycine, and lysine, while the overall amino acid content was lower than fishmeal and soybean meal (Khoshnevisan et al., 2019).

Apart from their suitable amino acid profile, both MOB and HOB are characterized by a variety of nutritional properties that render them compelling for high quality food/feed production. For instance, MOB and HOB are known to accumulate PHB, at contents up to 68 and 89% w/w, respectively depending on the cultivation conditions (Khosravi-Darani et al., 2013). This increases the value of the product as there is proof that PHB can act as prebiotics (De Schryver et al., 2010). Furthermore, MOB biomass is rich in vitamins. Methanotroph-dominated MP products contain nicotinic acid (130 mg/kg), riboflavin (73 mg/kg), inositol (30 mg/kg), thiamin (12 mg/kg), vitamin B12 (1.7 mg/kg) and biotin (2.8 mg/kg) (Silverman, 2015). Other interesting features include the presence of carotenoids in MOB biomass that belong to the group of xanthophylls (Leadbetter and Foster, 1958), while the accumulation of carotenoids in HOB cells has also been reported (Davis et al., 1969). MOB can also be a source of unsaturated fatty acids, and macronutrients such as potassium (K), magnesium (Mg), and iron (Fe) (Kuźniar et al., 2019). It is worth noting that these two bacterial groups have different properties. For instance, MOB contain unsaturated acids, while HOB can accumulate very high amounts of PHB (Khosravi-Darani et al., 2013). Therefore, the combination of MOB and HOB can substantially increase the value of the final product.

Another important parameter to consider is the digestibility of the contained amino acids, where MOB and HOB present interesting results. It was found that the proteins contained in HOB biomass are of higher quality than plant proteins in terms of digestibility, while they are inferior to meat proteins (Volova and Barashkov, 2010). Specifically, Volova and Barashkov (2010) found that, unlike other protein sources, a big fraction of the proteins contained in HOB biomass (37–39%) are structural proteins that are less available to proteases and therefore are not well-digested. The corresponding value for meat proteins is 17% and for wheat bran proteins it is 58%. On the other hand, animals digest the amino acids that are contained in MOB biomass very efficiently. Feeding trials with mink (*Mustela vison*) revealed that the digestibility of EAA in MP consisting mainly of *Methylococcus capsulatus* ranges between 75 and 92%, when half of the protein in the feed originated from raw cod (*Gadus morhua*) fillet, and 50% from MP (Schøyen et al., 2005). A plethora of feeding trials presents promising results. For instance, feeding trials with Atlantic salmon (*Salmo salar*) revealed that feed containing up to 36% of MOB-based MP performs as well as fishmeal (Aas et al., 2006). Similarly, when 10% of soybean contained in conventional broiler feed was substituted by biomass from *Methylococcus capsulatus* and *Methylomonas albus*, there were no significant differences from the control in growth, efficiency of food conversion or nitrogen retention, while the concentrations of amino acids and uric acid in the plasma were not affected either (D’Mello, 1973). Finally, a *Methylococcus capsulatus*-dominated MP diet was found to positively impact the gastrointestinal health of salmonids (Romarheim et al., 2011) and mice (Kleiveland et al., 2013).

### Reality Check: Current Knowledge Gaps and Limitations

Currently, some knowledge gaps need to be filled to efficiently engineer the MP production systems aiming at high productivity of a high-quality, cost-competitive product. For instance, a variety of cultivation modes can be used for MP production, such as batch, fed-batch or continuous. Matassa et al. (2016) found that the volumetric productivity of HOB biomass, as well as the yields, substantially increase (2.8–4.8 times) through the cultivation in continuous mode, in comparison to the sequential batch mode. Similarly, D’Mello (1973) found that the content of a variety of amino acids (aspartic acid, glutamic acid, valine, leucine, tyrosine, phenylalanine, lysine, arginine, methionine, and cystine) was higher when *Methylococcus capsulatus* and *Methylomonas albus* were grown on continuous mode, compared to their cultivation in batch mode. Even though the continuous cultivation mode increases the risk of contamination in the case of pure cultures or synthetic communities, the combination of MOB and HOB can enable the removal of “leaked” metabolic byproducts (e.g., carboxylic acids), thereby eliminating the available niches and contributing to system stability (*Co-Cultivation of MOB and HOB for Microbial Protein Production Could Enable System Stability and Higher Resource Utilization Efficiency*). In addition, the nucleic acid content of bacterial cells increased at higher growth rates. Specifically, Volova and Barashkov (2010) found that the nucleic acid content of *S. carboxydohydrogena* Z1062 cells increased from *ca.* 6% to *ca.* 9% when the dilution rate increased from 0.05 to 0.25 h^−1^, due to the intensification of RNA synthesis. Even though this approach resulted in increased protein productivity and lower carbohydrate content (which are favorable for a MP product with high protein content) the increase of RNA content lowers the product quality. Consequently, the cultivation mode is an important aspect, because of a correlation between the increase in protein productivity and the increase in nucleic acid content. This is particularly important when considering the possibility for gout or kidney stone formation when consuming high nucleic acid content food/feed ingredients (El Ridi and Tallima, 2017). This could decrease the overall quality of the product. Therefore, it can be concluded that many tools can aid at steering the quality of the product while the tradeoff between quantity and quality should be carefully assessed.

Other parameters that specifically affect the growth of organisms that consume carbon and energy sources from the gas phase should be carefully selected. For instance, it was demonstrated that the selection of gas mixtures affects the protein content and productivity of mixed MOB and HOB co-cultures (Acosta et al., 2019). This is important considering that the MP production process proposed here is integrated with biogas production *via* anaerobic digestion, which is prone to variable product composition (e.g., variating ratios of CH_4_:CO_2_, potential presence of H_2_S). A carefully selected synthetic co-culture, composed of community members with complementary characteristics could enable higher adaptability to these potentially variable inputs and therefore contribute to overall system stability (Bothe et al., 2002). Furthermore, it was shown that the presence of H_2_S in the biogas affects the growth and nutritional quality of *Methylocapsa acidiphila* (Xu et al., 2020). The composition of the cultivation medium is also important, as it has been reported that the addition of 100 μg Cu^2+^/L to NMS medium significantly affects the cell density of MOB since Cu is a key element for the methanotrophic metabolism (Semrau et al., 2018). Another challenge for the industrial production of MOB and HOB concerns the mass transfer limitation due to the low solubility of carbon and/or energy sources (i.e., CH_4_ and H_2_) in water. Some approaches have proven to efficiently increase the availability of substrates. For instance, it was shown that HOB grow more efficiently when the gas is continuously fed to maintain constant pressure close to atmospheric (Cohen and Burris, 1955). Similarly, a continuous methane supply is required to maintain sufficient amounts of soluble methane and avoid the reduced availability to the cells (Tikhomirova and But, 2021). However, this leads to big volumes of methane needed. The required volume can be minimized by adjusting the reactor geometry (e.g., ratio height-to-inner diameter), using gas-permeable membranes, agitation, recirculation and/or pre-mixing methane with the cultivation medium (Tikhomirova and But, 2021). Furthermore, the addition of 10% v/v silicon oil in a two-phase partitioning bioreactor has resulted in 330% increased growth rate of *Methylosinus sporium* (Ordaz et al., 2014). Similar results were observed during the cultivation of *Methylosinus trichosporium* OB3b where the addition of 5% paraffin resulted in *ca.* 7 times higher biomass (14 g_CDW_/L) compared to no paraffin addition (Han et al., 2009). This phenomenon was attributed to the increased methane dissolution rate achieved by the addition of paraffin as well as the potential affinity of *M. trichosporium* OB3b to paraffin. Furthermore, the reactor design plays a catalytic role in the substrate supply. Loop bioreactors have been used to assure efficient methane supply to methanotrophs, through the increased gas flow in the reactor (Petersen et al., 2017), while further reactor optimization with high-performance agitation achieved a gas transfer coefficient (k_L_a) of 2,970 h^−1^ resulting in the production of 91 g_CDW_/L (Tanaka et al., 1995). These values are 2.5 and 3 times higher than the k_L_a and biomass concentration achieved in the U-Loop reactor (1,200 h^−1^ and 30 g_CDW_/L) (Eriksen et al., 2009; AIChE, 2014). Finally, Wendlandt et al. (2005) achieved 65 g/L biomass concentration of *Methylocystis* sp. using pressure bioreactors due to the increased methane solubility. However, the cost-efficiency and safety of this approach are still disputed. Bubble-free membrane bioreactors have been proposed as a promising approach to produce MP from MOB, since they eliminate the possibility of creating an explosive atmosphere and result in 33% higher N yield compared to conventional reactors relying on bubbling (from 5.2 to 6.9 g_VSS_/g_NH3_) (Valverde-Pérez et al., 2020). Therefore, process and reactor design parameters need to be thoroughly investigated and carefully assessed to establish the optimal approach for MP production using MOB and HOB.

Consequently, even though the production of MOB and HOB for MP as food/feed ingredient seems promising, there are still some challenges that need to be overcome before their large-scale production on recovered resources can be realized.

## Conclusion

In this manuscript, we investigated if co-cultivation of HOB and MOB can be beneficial for MP production. The ten best combinations of HOB and MOB showed synergistic effects, with growth in co-cultures outperforming growth in axenic culture for six out of ten combinations. The combination of MOB and HOB resulted in up to 3.8 times higher protein concentration and 6.1 times higher EAA content compared to pure cultures, while the EAA profile of the (co-)cultures was comparable to common food ingredients. The most promising combination in terms of protein concentration and EAA profile was *Methyloparacoccus murrelli* LMG 27482 *and Cupriavidus necator* LMG 1201. Microbial protein from *M. murrelli and C. necator* requires 27–67% less quantity than chicken, whole egg and tofu, while it only requires 15% more quantity than the amino acid-dense soybean to cover the needs of an average adult. Hence, the combination of MOB and HOB can enable enhanced carbon recovery from biogas, and contribute to the development of more sustainable food/feed production systems.

## Data Availability

The datasets presented in this study can be found in online repositories. The names of the repository/repositories and accession number(s) can be found below: https://www.ncbi.nlm.nih.gov/nuccore/MT625930, https://www.ncbi.nlm.nih.gov/nuccore/MT625931, https://www.ncbi.nlm.nih.gov/nuccore/MT625932, https://www.ncbi.nlm.nih.gov/nuccore/MT625933, https://www.ncbi.nlm.nih.gov/nuccore/MT625934, https://www.ncbi.nlm.nih.gov/nuccore/MT625935, https://www.ncbi.nlm.nih.gov/nuccore/MT625936, https://www.ncbi.nlm.nih.gov/nuccore/MT625937, https://www.ncbi.nlm.nih.gov/nuccore/MT625938, https://www.ncbi.nlm.nih.gov/nuccore/MT625939, https://www.ncbi.nlm.nih.gov/nuccore/MT625940, https://www.ncbi.nlm.nih.gov/nuccore/MT625941, https://www.ncbi.nlm.nih.gov/nuccore/MT625942, https://www.ncbi.nlm.nih.gov/nuccore/MT625943, https://www.ncbi.nlm.nih.gov/nuccore/MT625944, https://www.ncbi.nlm.nih.gov/nuccore/MT625945, https://www.ncbi.nlm.nih.gov/nuccore/MT625946, https://www.ncbi.nlm.nih.gov/nuccore/MT625947, https://www.ncbi.nlm.nih.gov/nuccore/MT625948, https://www.ncbi.nlm.nih.gov/nuccore/MW287569, https://www.ncbi.nlm.nih.gov/nuccore/MW287568, https://www.ncbi.nlm.nih.gov/nuccore/MW287570, https://www.ncbi.nlm.nih.gov/nuccore/MZ701924, https://doi.org/10.6084/m9.figshare.14776272.v1, https://doi.org/10.6084/m9.figshare.14776263.v1, https://github.com/CMET-UGent/Kerckhof_Sakarika_2021.
