# Supplementary material for: From Biogas and Hydrogen to Microbial Protein Through Co-Cultivation of Methane and Hydrogen Oxidizing Bacteria
Source: Front Bioeng Biotechnol. 2021 Aug 30;9:733753. doi: 10.3389/fbioe.2021.733753 (PMC8435580; doi:10.3389/fbioe.2021.733753)
Supplement: Supplementary file 1 [file Table.DOCX]

Supplementary Material for “*From biogas and hydrogen to microbial protein through co-cultivation of methane and hydrogen oxidizing bacteria*”

**Frederiek-Maarten Kerckhof^1,2^**^†^**, Myrsini Sakarika^1,2^**^†^**, Marie Van Giel^1^, Maarten Muys^3^, Pieter Vermeir^4^, Jo De Vrieze^1^, Siegfried E. Vlaeminck^1,3^, Korneel Rabaey^1,2^, Nico Boon^1,2*^**

^1^Center for Microbial Ecology and Technology, Faculty of Bioscience Engineering, Ghent University, Coupure Links 653, 9000 Gent, Belgium

^2^Center for Advanced Process Technology for Urban Resource Recovery (CAPTURE), Frieda Saeysstraat, 9052 Gent, Belgium

^3^Research Group of Sustainable Energy, Air and Water Technology, Department of Bioscience Engineering, University of Antwerp, Groenenborgerlaan 171, 2020 Antwerpen, Belgium

^4^Laboratory of Chemical Analysis, Department of green chemistry and technology, Faculty of Bioscience engineering, Ghent University, Valentin Vaerwyckweg 1, 9000 Ghent, Belgium

^†^These authors have contributed equally to this work and share first authorship

*** Correspondence:**Nico Boon
[nico.boon@ugent.be](mailto:nico.boon@ugent.be)nico.boon@UGent.be

# Supplementary Materials and Methods

## Enrichment of hydrogen oxidizing bacteria

To obtain diverse inocula, rich in HOB, two cycles of enrichments were performed, as described by Ehsani et al., (2019). In brief, a soil sample was obtained from the surface soil as well as 0.1 m depth from the surface layer of the Almoeseneie forest in Gontrode, Belgium (50°58’N 3°49’E) (Vanhellemont et al., 2014). The soil samples were initially enriched under a sequential batch regime, where H_2_ and O_2_ were produced through water electrolysis. After 10 cycles of the initial batch enrichment, the enrichment regime was changed to continuous, using four 1 L glass reactors, with a working volume of 0.4 L (Ehsani, 2020). The H_2_ and O_2_ were produced through water electrolysis and were mixed with 1M NaHCO_3_ and circulated in the shared headspace of all reactors. Fresh medium for chemolithotrophic growth (DSMZ medium 81) was provided using a syringe pump at a flow rate of 3 mL/min, resulting in a hydraulic retention time (HRT) of 120 h.

Next, four opaque penicillin bottles with a final volume of 120 mL containing mineral medium were inoculated (10% v/v) with the enriched culture to result in 20 mL final volume, with a headspace composition of 2% O_2_, 10% H_2_, 10% CO_2_ and 78% N_2_. The penicillin bottles were incubated at 120 rpm and 28°C. The first two cultivation cycles were performed by transferring 50% of the cultures (10 mL) to fresh medium (10 mL) to achieve a final volume of 20 mL. When consumption of gases was noted (indicated by pressure drop), the headspace was refreshed. The following transfers and headspace refreshments were performed every 15 hours for the following 1 month. When all four cultures showed active H_2_ oxidation (indicated by gas consumption) the headspace was refreshed every 5 hours and the cultures were transferred every 10 hours. This period lasted 3 months, at the end of which, a sample was taken for the isolation campaign.

## Optical density

The cell growth during the co-cultivation tests was monitored by measuring the optical density (OD) at 600 nm, using a Tecan Infinite® M200 Pro microplate reader (Tecan™, Männdorf, Switzerland) with Tecan i-control reader and analysis software. After removing the plates from the gastight jars, the plates were brought at 28°C into the plate reader for immediate analysis. In cases where flocks were present, the plate was shaken shortly (*c.a.* 5 sec) to dissolve the flocks. A lid-correction was applied in the software, and plates were inspected for condensation before measurements.

## DNA extraction

Pellets of liquid culture broth were prepared from 2 mL of actively growing culture by centrifuging for 5 min at 21130 g. When the pellet was not deemed voluminous enough, a volume of 5 to 10 mL culture medium was first centrifuged for 5 min at 7745 g and the pellet was redissolved in 2 mL PBS or culture medium (AMS, NMS or DSMZ medium 81, depending on the kind of bacteria) and transferred to a 2 mL tube, and again centrifuged for 5 minutes at 21130 g and the supernatant was removed. The de-watered pellets could be stored in the freezer (-20°C) for a couple of days.

DNA extraction was performed as described before by De Rudder and colleagues (De Rudder et al., 2020): 200 mg of glass beads (Sartorius, Goettingen, Germany) and 1000 μL of lysis buffer were added to the pellets. Lysis buffer is composed out of 100 mM Tris pH 8, 100 mM 2 x NaEDTA, 100 mM NaCl, 1% polyvinylpyrrolidone (PVP40) and 2% sodium dodecyl sulphate (SDS) in water. The tubes were transferred to the PowerLyzer™ 24 Homogenizer (Qiagen, Hilden, Germany) to disrupt the cells at 2000 rpm for 5 minutes. Next, the samples were centrifuged for 5 minutes at 21,130 g. The supernatant from the samples was transferred to an microcentrifuge tube containing 500 μL of an phenol:chloroform:isoamilic alcohol pH 7 mixture. The tubes were inverted and centrifuged for 1 min at 21,130 g. The aqueous (upper) phase of the sample was then transferred to a tube containing 700 μL of chloroform. The tubes were inverted and centrifuged for 1 min at 21,130 g. A volume of 450 μL of the supernatant was transferred to an Eppendorf tube containing 45 μL sodium acetate and 500 μL isopropanol. Mixing was done by inverting the tubes. The samples were stored at -20°C for at least one hour and after this time, the tubes were centrifuged at 18,210 g at 4°C for 30 min. The pellet was then dried by pouring the supernatant off, and leaving the tube inverted on the bench for max. 10 minutes. After this step, 50 μL of TE 1x was added to dissolve the pellet. The TE buffer is composed of 10 mM Tris at pH 8.0 (adjusted with HCl) and 1 mM EDTA).

Quality of the DNA extract was evaluated by running the DNA extract for 30 min at 100V on a 2% agarose gel and was visualized with Ethidium Bromide using a UV transilluminator. The concentration was (where needed) determined using the QuantiFluor® dsDNA System (Promega, USA), according to the manufacturer’s instructions.

## Sanger sequencing

To identify isolates and track purity of the strains, near full-length amplicon of the 16S rRNA gene of individual isolates was purified, and sent out for Sanger sequencing (LGC Genomics Gmbh, Berlin). Near full-length amplicon was obtained using the primer set 27F/1492R (Weisburg et al., 1991) with sequences AGAGTTTGATCMTGGCTCAG/ TACGGYTACCTTGTTACGACTT respectively (5’-3’). PCR amplification was performed on a Bio-Rad T100 Thermo Cycler (Bio-Rad laboratories Inc., Hercules, California, USA), and all reactions were carried out in total volumes of 25 µL of mixture. The mixtures contained 2.5 µL 10x TAQ buffer + KCl – MgCl2, 1.5 µL MgCl2, (25 mM), 0.5 µL dNTP (10 mM of each), 0.5 µL Forward Primer (10 µM), 0.5 µL Reverse Primer (10 µM), 0.125 µL, Taq polymerase (5 U/µL), 0.065 µL Bovine serum albumin (BSA) (20 mg/mL), 18.31 µL PCR water, 1 µL of the template DNA. In total 30 cycles were executed after an initial denaturation of 7 minutes at 95°C and then 30x [1' 94°C, 1' 55°C, 2' 72°C] with a final elongation of 10 minutes at 72°C. The sequences were classified with the Ribosomal Database Project (RDP16; 80% confidence threshold), SILVA nr release 132 and NCBI Blast. Taxonomic assignments were compared, and the most appropriate classification was assigned to the samples.

## Selection of gas composition

The gas composition on the serum vial experiments was selected based on:

the theoretical required ratio of H_2_/CO_2_ for CO_2_ fixation is 4 (Yu et al., 2013) (this study: 4)

the ratio of H_2_/O_2_ during water electrolysis is 2 (this study: 1.5)

the typical concentration of biogas is 60% CH_4_ and 40% CO_2_, resulting in a ratio CH_4_/CO_2_=1.5 (this study: 1.0)

the O_2_/CO_2_ ratio of 2 was selected based on Yu et al. (2013) (this study: 2.6).

Given the difficulty to manipulate gases, and the large number of ratios that we needed to abide by, the final concentrations deviated from the optimal ones.

## Total protein quantification

The protein analysis was performed using the DC™ Protein Assay (Bio-Rad Laboratories Inc., Hercules, USA). To form a biomass pellet, 5 mL of liquid culture was centrifuged at 7,745 g, the supernatant was removed, and the pellets were stored at -80°C. Next, 100 μL MQ water was added to each tube. The tubes were vortexed and stored at -20°C for two days. A standard curve was made by using of Pierce™ Bovine Serum Albumin (BSA; Thermo Fisher Scientific, Waltham, Massachusetts, USA), and the total protein was quantified by measuring the absorbance at 750 nm. Analysis was performed in triplicate for each sample.

## Phylogenetic tree inference

The sanger read data was aligned using the on-line SILVA SINA alignment & classification tool (Pruesse et al., 2012) (<https://www.arb-silva.de/aligner/>) and minimized using BioEdit. Phylogeny was inferred from this alignment using RAxML 8.2.11 (Stamatakis, 2014) using the Gamma model of rate heterogeneity, with a ML estimate of the alpha parameter. The BFGS method was used to optimize GTR substitution matrix rate parameters. First 1000 rapid bootstraps were executed, followed by a ML search and a thorough tree optimization. Tree visualization and annotation was performed in iTol (Letunic and Bork, 2021) (<https://itol.embl.de>).

## Calculations

Estimations were made for the expected cell densities as well as for the protein concentrations of the MOB and HOB combinations, based on the average results of the biological and technical replicates of the pure cultures. An assumption of equal contribution of the MOB and HOB was considered. An example for the calculation of the protein concentration is given below (Equation 1):

| $P_{combination} \left[ \frac{mg}{L} \right]= \frac{P_{MOB} \left[ \frac{mg}{L} \right]}{2}+\frac{P_{HOB} \left[ \frac{mg}{L} \right]}{2}$ | Equation 1 |
| --- | --- |

# Supplementary Results

## Process integration is key for near-complete resource valorization leading to lower environmental footprint

To efficiently valorize the resources that are used as inputs in the proposed process (**Figure 1**), process integration is essential. Considering that the produced biogas is composed of 60%_mol_ CH_4_ and 40%_mol_ CO_2_ and the following stoichiometry for the growth of MOB (Nielsen and Villadsen, 1994) (Eq. 1) and HOB (Ishizaki and Tanaka, 1990) (Eq. 2) as well as water electrolysis (Eq. 3), the process outputs were estimated:

| CH_4_ + 1.5 O_2_ + 0.10 NH_3_ 🡪 0.52 CH_1.8_O_0.5_N_0.2_ + 0.48 CO_2_ + 1.7 H_2_O | Eq. 1 |
| --- | --- |
| H_2_ + 0.46 O_2_ + 0.035 CO_2_ + 0.0066 NH_3_🡪 0.035 CH_1.7_O_0.5_N_0.2_ + 0.98 H_2_O | Eq. 2 |
| 2 H_2_O🡪 2 H_2_ + O_2_ | Eq. 3 |

To execute the required calculations, the following assumptions are made:

1. Only stoichiometry is taken into account, *i.e.*, mass transfer limitations and potential gas use inefficiencies are not considered
2. Ammonia production through anaerobic digestion is waste-specific, and therefore, is not considered here
3. No competition for O_2_ utilization is considered (*i.e.* the available O_2_ can be utilized at equal rates and/or quantities by the MOB and HOB)
4. The HOB consume the CO_2_ from biogas as well as the CO_2_ metabolically produced from MOB

Our analysis shows that O_2_ needs to be supplemented to fully utilize the H_2_ produced *via* water electrolysis (the summary of the calculation is presented in **Supplementary Table 9**). This could be solved by air supplementation. The case where no air is supplemented results in MOB:HOB ratio of 5.0:1.0 wt. and a biomass yield of 0.31 kg_biomass_/kg_CODadded_. Air supplementation would result in MOB:HOB ratio of 2.7:1.0 wt. due to 86% higher quantity of HOB biomass, and would enable14% increased biomass yield (0.35 kg_biomass_/kg_CODadded_). In the latter case, the off-gas contains CO_2_ and N_2,_ at a ratio of 4.9 mol N_2_ per mol CO_2_. In this case, including N_2_-fixing HOB (Hu et al., 2020) and/or N_2_-fixing MOB (Khmelenina et al., 2018) would add value, since the nitrogen utilization of the overall process would be increased. Nevertheless, further investigations are required to validate this approach, since the lower growth rates of N_2_-fixers could compromise the productivity and the overall economics of the proposed process. The addition of air could also lead to a process with a lower carbon footprint, arising from the higher HOB biomass generation. Specifically, it results in 20% lower CO_2_ emissions (2.4 kg_CO2_/kg_biomass_) compared to the base case (3.0 kg_CO2_/kg_biomass_). Finally, another advantage of the approach proposed here is that the H_2_O generated can be harvested from the off-gas vapors and be redirected to water electrolysis, therefore, minimizing the water footprint of the process. In the base-case scenario (no air supplementation) 0.82 mol H_2_O are metabolically generated per mol H_2_O added, while in the case of air supplementation this number amounts to 1.3. These stoichiometric calculations indicate that process integration and careful design can increase the process efficiency and reduce the overall environmental footprint.

## Comparison of quality of microbial protein from MOB and HOB to food and feed ingredients

To assess the nutritional quality of each (co-)culture for other protein sources, the EAA profiles were compared with common food ingredients (**Figure 5**). The amount of each food ingredient needed to meet the nutritional requirements of an average adult weighing 62 kg (Walpole et al., 2012) is 121 g wet weight (g_ww_) of soybean, 191 g_ww_ of chicken, 218 g_ww_ of whole egg and 421 g_ww_ of tofu (**Supplementary Table 9**). Pure cultures and co-cultures that cover these needs with less quantity than chicken meat (191 g_ww_) are MOB6∙CNEC (139 g_ww_); CNEC (147 g_ww_); HOB18 (151 g_ww_); MOB8∙HOB13 (162 g_ww_) and MOB6∙HOB16 (179 g_ww_). Furthermore, the product that can cover the needs with less quantity than whole eggs (218 g_ww_) is derived from the cultures XAUT (197 g_ww_) and HOB15 (194 g_ww_). Lastly, the strains and combinations that cover the needs of a 62 kg adult with less quantity than tofu from soybean (421 g_ww_) are MOB1 (244 g_ww_); MOB8∙HOB15 (332 g_ww_) and MOB8∙HOB18 (369 g_ww_).

In comparison to tofu, all cultures were richer in histidine, lysine, phenylalanine, threonine, valine, leucine and isoleucine, while only MOB6∙HOB16 was poorer in phenylalanine (**Figure 5**; **Supplementary Table 9**). Compared to soybean, all cultures had lower content in histidine, lysine, phenylalanine, threonine, leucine and isoleucine, while only MOB6∙CNEC and MOB8∙HOB13 presented a higher valine content. In comparison to raw chicken, all cultures had higher valine, leucine and isoleucine content (for the latter two, the values of MOB6∙HOB16 are comparable with raw chicken), while only HOB18 and CNEC had higher histidine and phenylalanine content. Regarding lysine, only HOB18 had a comparable content, while only MOB6∙CNEC and HOB18 had higher threonine content compared to raw chicken. All cultures have higher histidine, lysine, valine, leucine and isoleucine content compared to whole egg. The CNEC, HOB18, MOB8∙HOB13 and MOB6∙HOB16 cultures have higher threonine content compared to whole egg, while only HOB18 and CNEC have higher phenylalanine content. Finally, when these cultures are compared to fishmeal, all were poorer in histidine, lysine and threonine, while HOB18 had higher phenylalanine content, MOB6∙CNEC and MOB8∙HOB13 were richer in valine, MOB6∙CNEC and HOB18 had higher or comparable leucine content while when comparing the isoleucine MOB6∙CNEC had a comparable content.

# Supplementary Figures and Tables

## Supplementary Figures


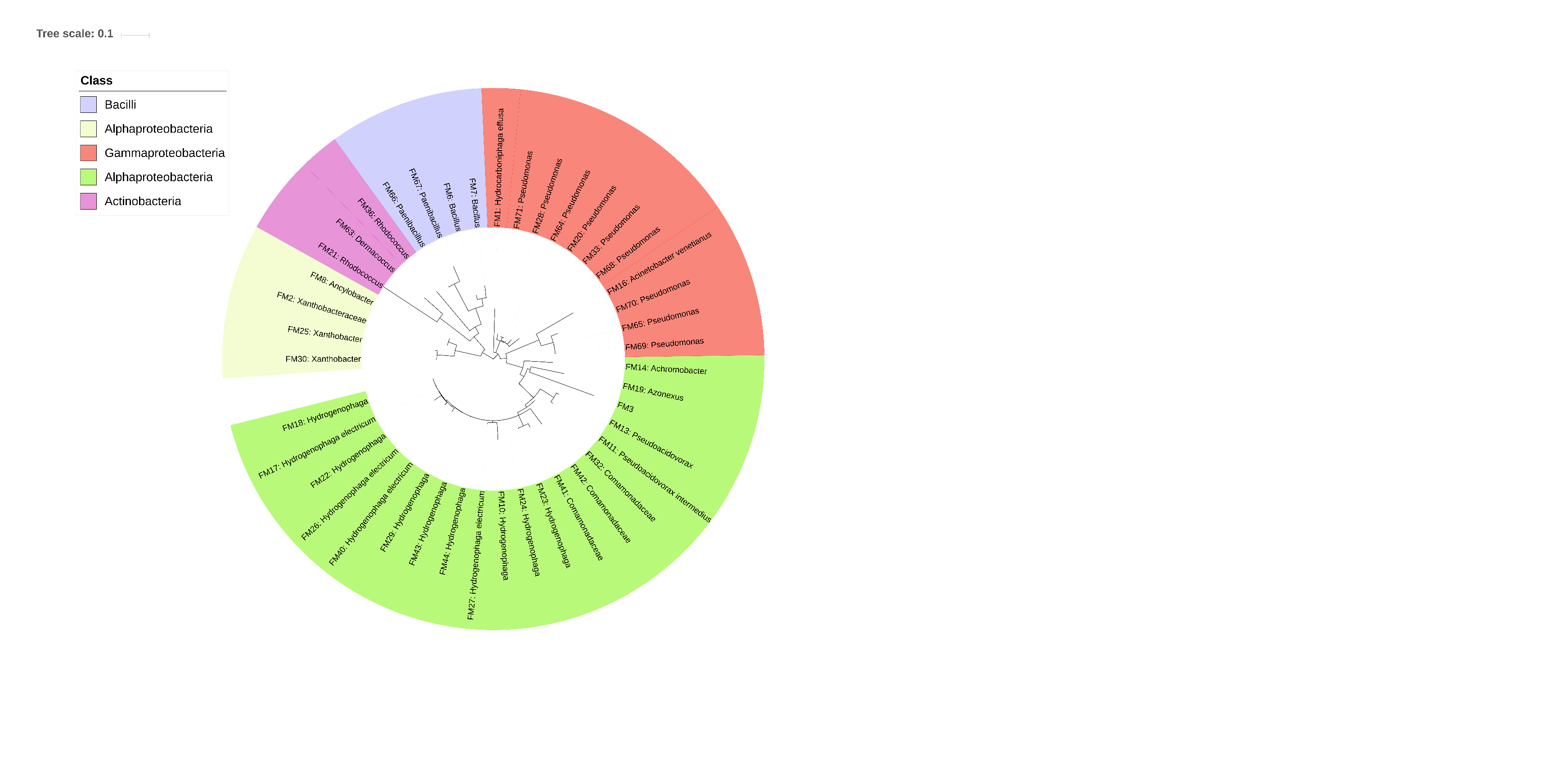


Supplementary Figure 1. Phylogenetic tree of full-length (27f/1492r) 16S rRNA gene of the isolates (SINA alignment, RAxML GTR+GAMMA, 1000 rapid bootstraps, thorough tree optimization was executed, with a final likelihood of -9085.196276)


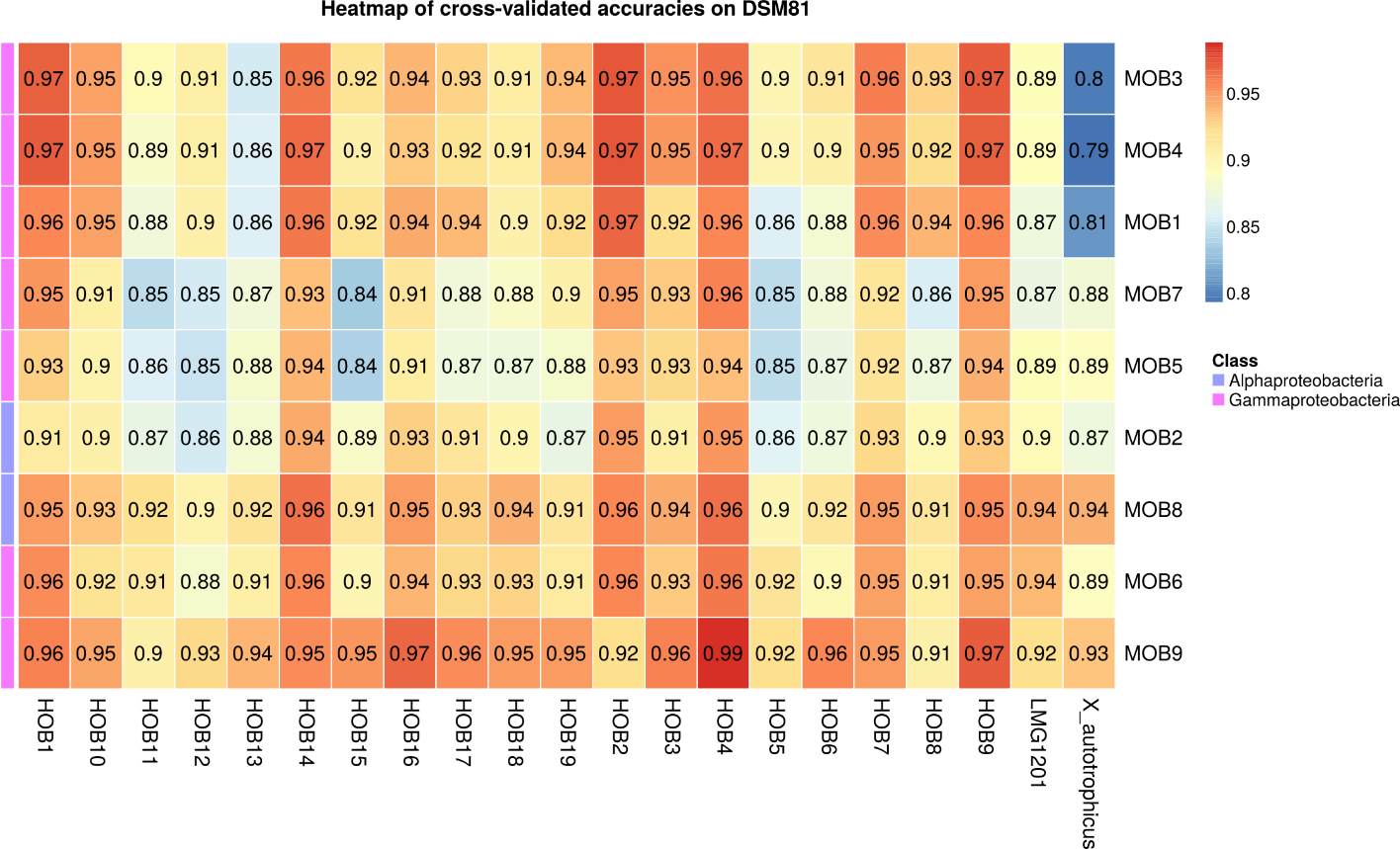


Supplementary Figure 2. Accuracy values for random-forest based prediction based upon flow cytometry data of the axenic cultures pre-grown on DSM81, that were trained into the combinations that were made.


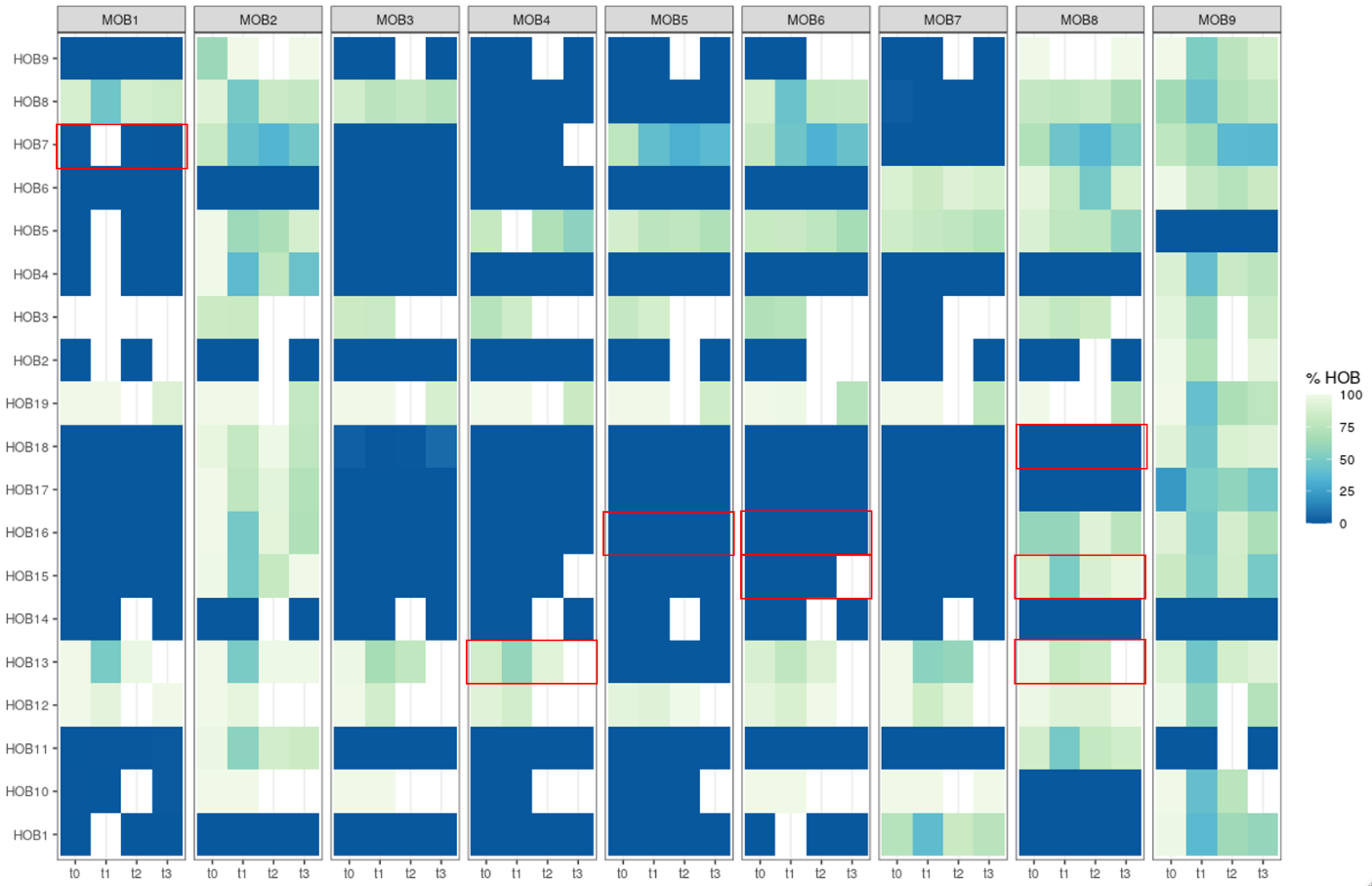


Supplementary Figure 3. Time-series population dynamics as percent of HOB cells in the total population inferred from pre-trained axenic pairwise models grown on DSM 81. If no data is shown, less than 1,000 cells were available and no inference could be reliably made, or model predictions failed. Combinations in red were the selected for further follow-up.


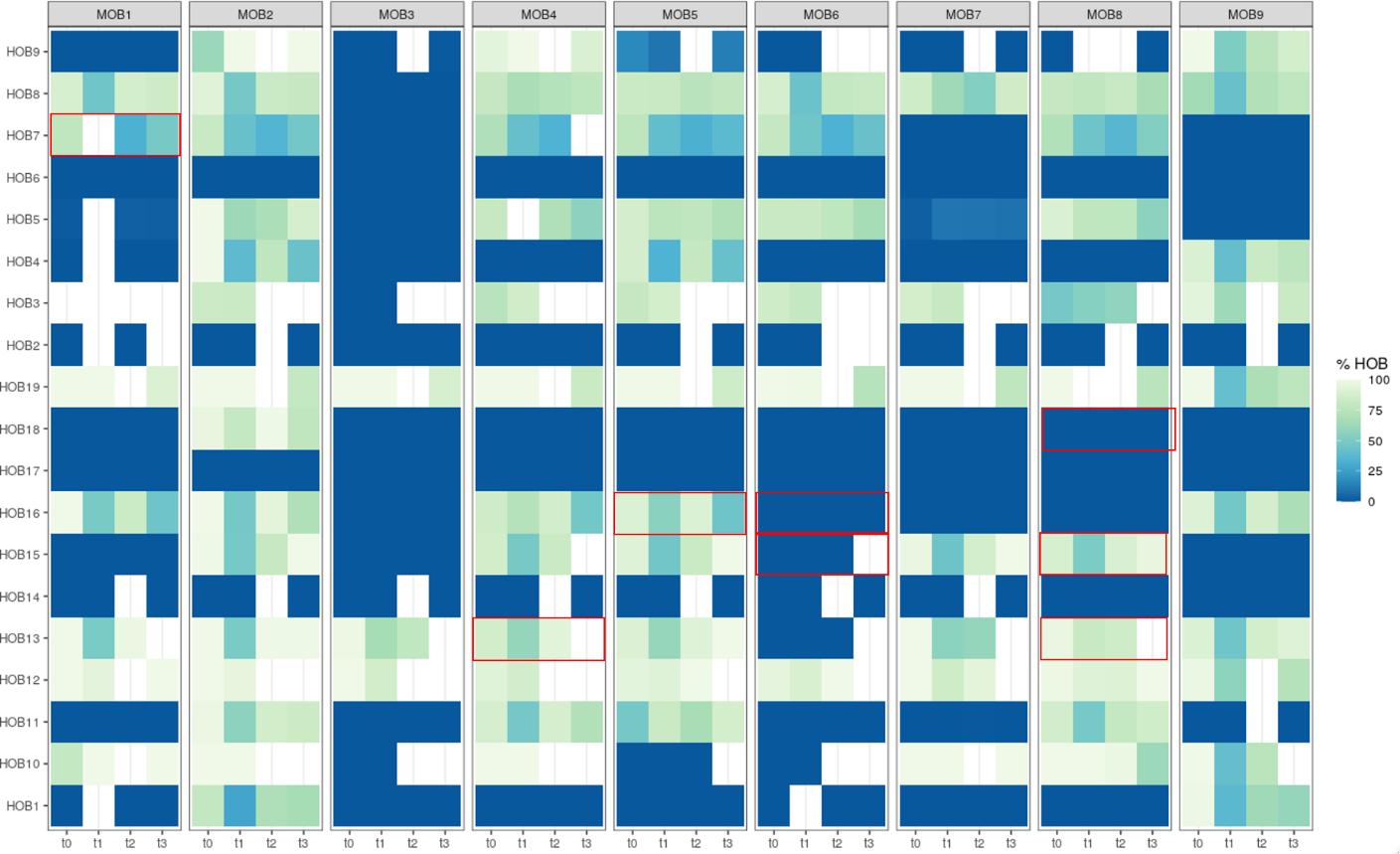


Supplementary Figure 4. Time-series population dynamics as percent of HOB cells in the total population inferred from pre-trained axenic pairwise models where the MOB were pre-grown on AMS. If no data is shown, less than 1,000 cells were available and no inference could be reliably made, or model predictions failed. Combinations in red were the selected ones.


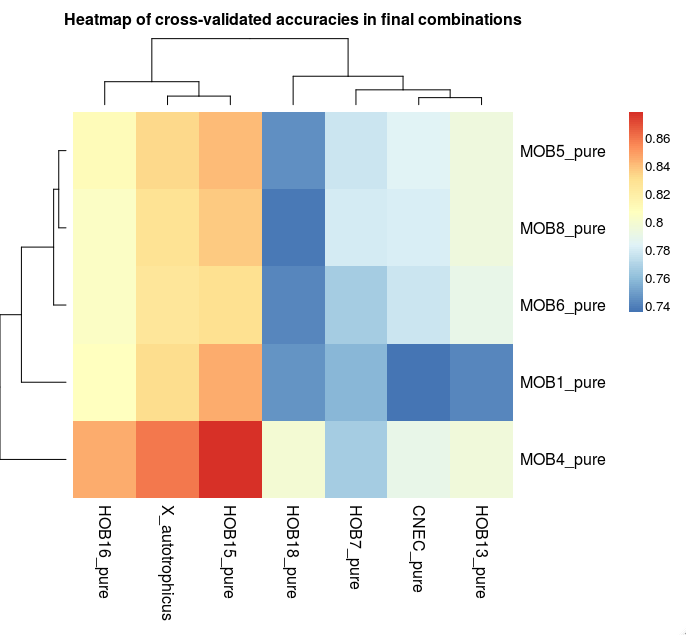


Supplementary Figure 5. Heat map of cross-validated accuracies on pre-trained axenic pairwise models grown on DSM 81 of the final combinations.


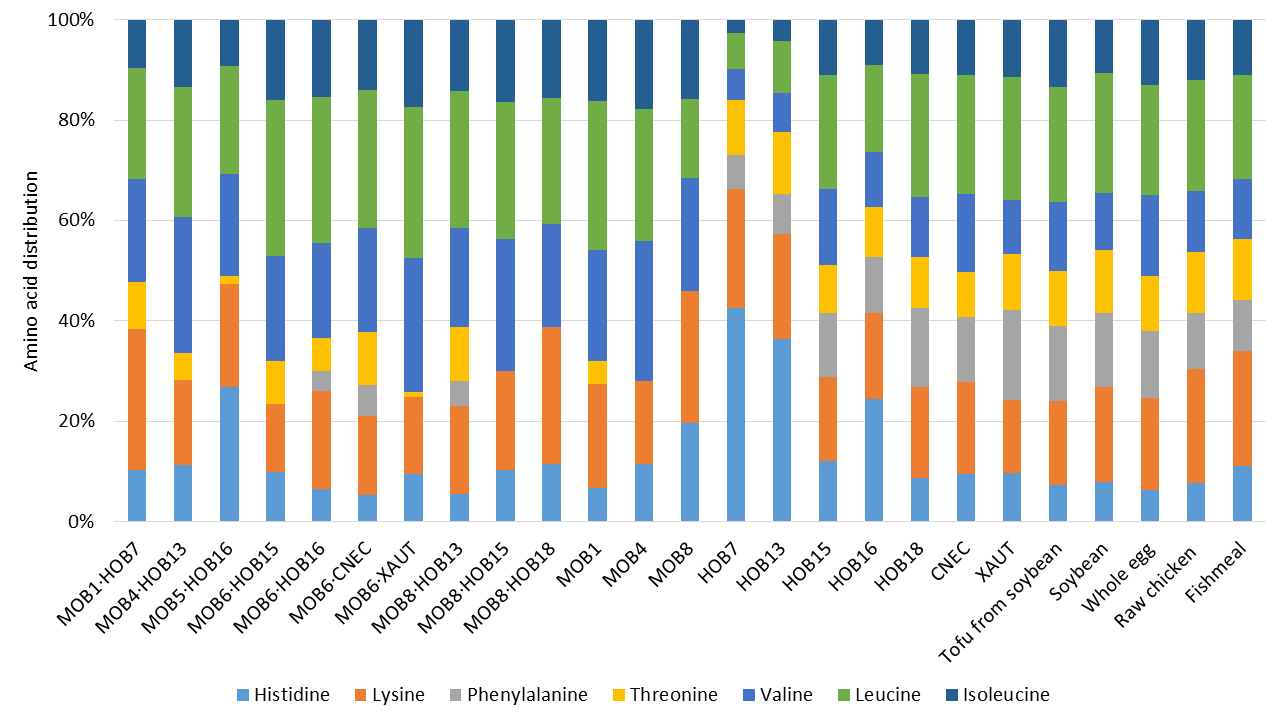


Supplementary Figure 6. Amino acid distribution of microbial biomass and various food and feed ingredients

## Supplementary Tables

**Supplementary Table 1**: Composition of medium DSMZ 81 (<https://www.dsmz.de/microorganisms/medium/pdf/DSMZ_Medium81.pdf>).

| Compound | Unit | Value |
| --- | --- | --- |
| NH_4_Cl | g L^-1^ | 1.0 |
| MgSO_4_ × 7 H_2_O | g L^-1^ | 0.5 |
| CaCl_2_ × 2 H_2_O | mg L^-1^ | 10.0 |
| MnCl_2_ × 4 H_2_O | mg L^-1^ | 5.0 |
| NaVO_3_ × H_2_O | mg L^-1^ | 5.0 |
| 5% NaHCO_3_ | g L^-1^ | 0.50 |
| Ferric ammonium citrate | g L^-1^ | 0.05 |
| Phosphate buffer | | |
| KH_2_PO_4_ | g L^-1^ | 2.3 |
| Na_2_HPO_4_ × 2 H_2_O | g L^-1^ | 2.9 |
| Trace elements | | |
| ZnSO_4_ × 7 H_2_O | mg L^-1^ | 0.50 |
| MnCl_2_ × 4 H_2_O | mg L^-1^ | 0.15 |
| H_3_BO_3_ | mg L^-1^ | 1.49 |
| CoCl_2_ × 6 H_2_O | mg L^-1^ | 1.00 |
| CuCl_2_ × 2 H_2_O | mg L^-1^ | 0.05 |
| NiCl_2_ × 6 H_2_O | mg L^-1^ | 0.10 |
| Na_2_MoO_4_ × 2 H_2_O | mg L^-1^ | 0.15 |
| Vitamins | | |
| riboflavin | mg L^-1^ | 0.005 |
| thiamine-HCl × 2 H_2_O | mg L^-1^ | 0.025 |
| nicotinic acid | mg L^-1^ | 0.025 |
| pyridoxine-HCl | mg L^-1^ | 0.025 |
| Ca-pantothenate | mg L^-1^ | 0.025 |
| biotin | μg L^-1^ | 0.05 |
| folic acid | μg L^-1^ | 0.1 |
| B_12_ | μg L^-1^ | 0.5 |

**Supplementary Table 2**: Composition of nitrate mineral salts (NMS) medium

| Compound | Unit | Value |
| --- | --- | --- |
| MgSO_4_ × 7 H_2_O | g L^-1^ | 1.0 |
| KNO_3_ | g L^-1^ | 1.0 |
| CaCl_2_ × 2 H_2_O | g L^-1^ | 0.15 |
| FeNaEDTA^*^ | mg L^-1^ | 0.05 |
| Trace elements | | |
| Na_2_EDTA^*^ × 2 H_2_O | mg L^-1^ | 0.5 |
| FeSO_4_ × 7 H_2_O | mg L^-1^ | 0.2 |
| H_3_BO_3_ | mg L^-1^ | 0.03 |
| CoCl_2_ × 6 H_2_O | mg L^-1^ | 0.02 |
| ZnSO_4_ × 7 H_2_O | mg L^-1^ | 0.01 |
| MnCl_2_ × 4 H_2_O | mg L^-1^ | 0.003 |
| NaMoO_4_ × 2 H_2_O | mg L^-1^ | 0.003 |
| NiCl_2_ × 6 H_2_O | mg L^-1^ | 0.002 |
| CuSO_4_ × 5 H_2_O | mg L^-1^ | 2.5 |
| Phosphate buffer | | |
| Na_2_HPO_4_ × 12 H_2_O | g L^-1^ | 0.717 |
| KH_2_PO_4_ | g L^-1^ | 0.272 |

*^*^*Ethylenediaminetetraacetic acid

Supplementary Table 3: Cell concentrations of the selected combinations after 6 days of co-cultivation in a microtiter plate as compared to the expected cell concentration of a 1:1 mixture of the axenic cultures that were taken along as controls.

| Combination | Measured cell concentration  (cells/mL) | Expected cell concentration (cells/mL) |
| --- | --- | --- |
| MOB1·HOB7 | 4.99 × 10^9^ | 1.25 × 10^9^ |
| MOB4·HOB13 | 3.54 x 10^7^ | 1.37 x 10^7^ |
| MOB5·HOB16 | 2.91 x 10^8^ | 2.37 x 10^7^ |
| MOB6·HOB15 | 1.96 x 10^8^ | 4.62 x 10^7^ |
| MOB6·HOB16 | 2.97 x 10^8^ | 2.63 x 10^7^ |
| MOB8·HOB13 | 9.75 x 10^7^ | 1.78 x 10^7^ |
| MOB8·HOB15 | 3.54 x 10^8^ | 4.73 x 10^7^ |
| MOB8·HOB18 | 4.11 x 10^7^ | 9.87 x 10^6^ |
| MOB6·CNEC | 7.09 × 10^9^ | 2.12 × 10^9^ |
| MOB6·XAUT | 4.91 x 10^7^ | 2.61 x 10^7^ |

Supplementary Table 4: Average measured and predicted total protein content of the selected combinations. The standard deviation is the propagated standard deviation of biological (n=3) and technical (n=3) replicates. The ratios of the averages higher than 1 are presented here in bold.

| Combination | Measured protein content (pg/cell) | Predicted protein content (pg/cell) | Ratio measured to predicted |
| --- | --- | --- | --- |
| MOB1·HOB7 | 0.475 ± 0.174 | 0.465 | 1.02 |
| MOB4·HOB13 | 0.157 ± 0.035 | 0.222 | 0.706 |
| MOB5·HOB16 | 0.109 ± 0.018 | 0.264 | 0.413 |
| MOB6·HOB15 | 0.103 ± 0.038 | 0.325 | 0.317 |
| MOB6·HOB16 | 0.038 ± 0.012 | 0.320 | 0.119 |
| MOB6·CNEC | 0.078 ± 0.062 | 0.364 | 0.215 |
| MOB6·XAUT | 0.093 ± 0.045 | 0.304 | 0.307 |
| MOB8·HOB13 | 0.241 ± 0.235 | 0.330 | 0.731 |
| MOB8·HOB15 | 0.109 ± 0.037 | 0.185 | 0.590 |
| MOB8·HOB18 | 0.242 ± 0.063 | 0.175 | 1.38 |

Supplementary Table 5: Average measured and predicted total protein concentration of the selected combinations. The standard deviation is the propagated standard deviation of biological (n=3) and technical (n=3) replicates. The ratios of the averages higher than 1 are presented here in bold. The asterisk indicates the significant values (^(1)^Welch t-test if normality assumptions were met, otherwise ^(2)^Wilcoxon rank-sum test on all observations, n=9, with a two-sided alternative hypothesis).

| Combination | Measured protein concentration (mg/L) | Predicted protein concentration (mg/L) | Ratio measured to predicted | p-value |
| --- | --- | --- | --- | --- |
| MOB1·HOB7 | 6.27 ± 1.76 | 5.34 ± 6.06 | **1.17** | 2.51 · 10^-1 (2)^ |
| MOB4·HOB13 | 3.41 ± 1.03 | 2.07 ± 1.29 | **1.65*** | 6.96 · 10^-5 (1)^ |
| MOB5·HOB16 | 4.79 ± 1.66 | 5.22 ± 1.06 | 0.92 | 9.68 · 10^-2 (1)^ |
| MOB6·HOB15 | 5.28 ± 1.90 | 6.40 ± 1.30 | 0.83 | 1.90 · 10^-1 (1)^ |
| MOB6·HOB16 | 2.00 ± 1.46 | 5.36 ± 1.21 | 0.37* | 8.81 · 10^-5 (1)^ |
| MOB6·CNEC | 30.5 ± 26.3 | 8.03 ± 1.74 | **3.79*** | 3.29 · 10^-2 (1)^ |
| MOB6·XAUT | 4.81 ± 1.60 | 5.28 ± 1.12 | 0.91 | 3.68 · 10^-1 (2)^ |
| MOB8·HOB13 | 3.05 ± 2.36 | 2.87 ± 1.28 | **1.06** | 2.75 · 10^-1 (1)^ |
| MOB8·HOB15 | 5.72 ± 1.55 | 5.24 ± 1.07 | **1.09** | 4.36 · 10^-1 (2)^ |
| MOB8·HOB18 | 3.43 ± 3.28 | 4.01 ± 1.26 | 0.86* | 1.68 · 10^-1 (1)^ |

**Supplementary Table 6**: Composition of essential and conditionally essential amino acids in the biomass of combined methane oxidizing bacteria (MOB) and hydrogen oxidizing bacteria (HOB) cultures as well as individual MOB and HOB strains. Tryptophan is not included since it is destroyed during acid hydrolysis. Common food and feed ingredients are included for comparison. The amino acid concentrations are presented in g/100 g_product_.

| Amino acids (g/100 g_product_) | Essential amino acids | | | | | | | Conditionally essential amino acids | | | Reference |
| --- | --- | --- | --- | --- | --- | --- | --- | --- | --- | --- | --- |
|  | **Histidine** | **Isoleucine** | **Leucine** | **Lysine** | **Phenylalanine** | **Threonine** | **Valine** | **Glutamine** | **Glycine** | **Proline** |  |
| MOB1·HOB7 | 0.255 | 0.237 | 0.541 | 0.685 | - | 0.234 | 0.503 | 0.967 | 0.585 | 0.224 | This study |
| MOB4·HOB13 | 0.266 | 0.311 | 0.607 | 0.394 | - | 0.123 | 0.632 | 0.997 | 0.687 | 0.284 |  |
| MOB5·HOB16 | 0.319 | 0.109 | 0.257 | 0.244 | - | 0.017 | 0.241 | 0.677 | 0.144 | 0.042 |  |
| MOB6·HOB15 | 0.326 | 0.531 | 1.030 | 0.455 | - | 0.283 | 0.691 | 1.879 | 0.864 | 0.444 |  |
| MOB6·HOB16 | 0.350 | 0.827 | 1.546 | 1.037 | 0.212 | 0.355 | 1.015 | 2.285 | 1.132 | 0.674 |  |
| MOB6·CNEC | 0.451 | 1.183 | 2.335 | 1.335 | 0.525 | 0.897 | 1.767 | 1.818 | 2.235 | 1.189 |  |
| MOB6·XAUT | 0.234 | 0.429 | 0.747 | 0.380 | - | 0.024 | 0.660 | 0.730 | 0.466 | 0.330 |  |
| MOB8·HOB13 | 0.382 | 1.369 | 1.369 | 1.214 | 0.355 | 0.745 | 1.369 | 1.369 | 1.369 | 1.369 |  |
| MOB8·HOB15 | 0.292 | 0.468 | 0.772 | 0.561 | - | - | 0.749 | 1.419 | 0.518 | 0.382 |  |
| MOB8·HOB18 | 0.304 | 0.412 | 0.655 | 0.714 | - | - | 0.542 | 0.821 | 0.300 | 0.245 |  |
| MOB1 | 0.254 | 0.615 | 1.129 | 0.793 | - | 0.172 | 0.845 | 0.773 | 0.690 | 0.546 |  |
| MOB4 | 0.223 | 0.346 | 0.506 | 0.320 | - | - | 0.540 | 0.553 | 0.077 | 0.249 |  |
| MOB8 | 0.204 | 0.163 | 0.163 | 0.272 | - | - | 0.232 | 0.287 | - | 0.074 |  |
| HOB7 | 0.987 | 0.063 | 0.163 | 0.551 | 0.156 | 0.253 | 0.148 | - | 0.319 | 0.364 |  |
| HOB13 | 0.998 | 0.118 | 0.279 | 0.572 | 0.218 | 0.338 | 0.214 | 0.051 | 0.384 | 0.409 |  |
| HOB15 | 0.699 | 0.638 | 1.321 | 0.968 | 0.748 | 0.548 | 0.879 | 1.313 | 0.852 | 0.733 |  |
| HOB16 | 0.679 | 0.254 | 0.480 | 0.481 | 0.308 | 0.279 | 0.307 | 0.186 | 0.374 | 0.387 |  |
| HOB18 | 0.782 | 0.968 | 2.197 | 1.623 | 1.396 | 0.904 | 1.069 | 1.711 | 1.222 | 0.956 |  |
| CNEC | 0.716 | 0.843 | 1.790 | 1.394 | 0.991 | 0.682 | 1.184 | 1.651 | 0.946 | 0.992 |  |
| XAUT | 0.731 | 0.864 | 1.832 | 1.100 | 1.341 | 0.843 | 0.817 | 1.939 | 0.885 | 0.898 |  |
| Tofu | 0.202 | 0.372 | 0.638 | 0.471 | 0.415 | 0.307 | 0.383 | N.A. | 0.317 | 0.419 | (Wang and Cavins, 1989) |
| Soybean | 0.926 | 1.232 | 2.812 | 2.204 | 1.727 | 1.463 | 1.328 | N.A. | 1.588 | 1.969 |  |
| Whole egg | 0.322 | 0.661 | 1.110 | 0.929 | 0.675 | 0.555 | 0.816 | N.A. | 0.437 | 0.493 | (American Egg Board, 2021) |
| Fishmeal | 1.214 | 1.202 | 2.244 | 2.486 | 1.089 | 1.326 | 1.296 | N.A. | 2.975 | 1.592 | (Raggi et al., 2019) |

*partially destroyed during acid hydrolysis

“-“ denotes that the values were below the quantification limit

N.A. = not available

**Supplementary Table 7**: Composition of essential and conditionally essential amino acids in the biomass of combined methane oxidizing bacteria (MOB) and hydrogen oxidizing bacteria (HOB) cultures as well as individual MOB and HOB strains. Tryptophan is not included since it is destroyed during acid hydrolysis. The amino acid concentrations are presented in mg/L_culture_.

| Amino acids (mg/L_culture_) | Essential amino acids | | | | | | | | Conditionally essential amino acids | | | | |
| --- | --- | --- | --- | --- | --- | --- | --- | --- | --- | --- | --- | --- | --- |
|  | **Histidine** | **Isoleucine** | **Leucine** | **Lysine** | **Phenylalanine** | **Threonine** | **Methionine*** | **Valine** | **Glutamine** | **Glycine** | **Proline** | **Cysteine*** | **Tyrosine*** |
| MOB1·HOB7 | 0.101 | 0.094 | 0.214 | 0.271 | - | 0.092 | - | 0.199 | 0.383 | 0.232 | 0.089 | - | - |
| MOB4·HOB13 | 0.066 | 0.077 | 0.150 | 0.098 | - | 0.031 | - | 0.157 | 0.247 | 0.170 | 0.070 | - | - |
| MOB5·HOB16 | 0.114 | 0.039 | 0.092 | 0.087 | - | 0.006 | - | 0.086 | 0.241 | 0.051 | 0.015 | - | - |
| MOB6·HOB15 | 0.104 | 0.169 | 0.328 | 0.145 | - | 0.090 | - | 0.220 | 0.599 | 0.275 | 0.141 | - | - |
| MOB6·HOB16 | 0.068 | 0.160 | 0.299 | 0.201 | 0.041 | 0.069 | - | 0.196 | 0.442 | 0.219 | 0.130 | - | - |
| MOB6·CNEC | 0.894 | 2.773 | 5.471 | 2.806 | 0.977 | 2.314 | - | 4.638 | 3.824 | 9.149 | 3.175 | - | - |
| MOB6·XAUT | 0.062 | 0.135 | 0.235 | 0.107 | - | 0.008 | - | 0.233 | 0.207 | 0.257 | 0.119 | - | - |
| MOB8·HOB13 | 0.073 | 0.187 | 0.362 | 0.232 | 0.068 | 0.142 | - | 0.261 | 0.568 | 0.434 | 0.214 | - | - |
| MOB8·HOB15 | 0.131 | 0.210 | 0.346 | 0.252 | - | - | - | 0.336 | 0.637 | 0.233 | 0.171 | - | - |
| MOB8·HOB18 | 0.146 | 0.198 | 0.315 | 0.343 | - | - | - | 0.260 | 0.394 | 0.144 | 0.118 | - | - |
| MOB1 | 0.029 | 0.069 | 0.127 | 0.089 | - | 0.019 | - | 0.095 | 0.087 | 0.078 | 0.061 | - | - |
| MOB4 | 0.035 | 0.054 | 0.079 | 0.050 | - | - | - | 0.084 | 0.086 | 0.012 | 0.039 | - | - |
| MOB5 | 0.074 | 0.004 | - | 0.019 | - | - | - | 0.090 | 0.021 | - | - | - | - |
| MOB6 | 0.089 | - | - | 0.017 | - | - | - | 0.073 | - | - | - | - | - |
| MOB8 | 0.062 | 0.050 | 0.050 | 0.083 | - | - | - | 0.071 | 0.087 | - | 0.022 | - | - |
| HOB7 | 0.069 | 0.004 | 0.011 | 0.039 | 0.011 | 0.018 | 0.007 | 0.010 | - | 0.022 | 0.025 | 0.179 | 0.023 |
| HOB13 | 0.069 | 0.008 | 0.019 | 0.040 | 0.015 | 0.024 | 0.007 | 0.015 | 0.004 | 0.027 | 0.028 | 0.180 | 0.023 |
| HOB15 | 0.253 | 0.231 | 0.478 | 0.350 | 0.270 | 0.198 | 0.023 | 0.318 | 0.475 | 0.308 | 0.265 | 0.610 | 0.129 |
| HOB16 | 0.174 | 0.065 | 0.123 | 0.123 | 0.079 | 0.071 | 0.016 | 0.079 | 0.048 | 0.096 | 0.099 | 0.448 | 0.057 |
| HOB18 | 0.167 | 0.207 | 0.471 | 0.348 | 0.299 | 0.194 | 0.016 | 0.229 | 0.367 | 0.262 | 0.205 | 0.367 | 0.120 |
| CNEC | 0.463 | 0.545 | 1.158 | 0.902 | 0.641 | 0.441 | 0.042 | 0.766 | 1.068 | 0.612 | 0.642 | 1.056 | 0.363 |
| XAUT | 0.158 | 0.187 | 0.397 | 0.238 | 0.290 | 0.182 | 0.015 | 0.177 | 0.420 | 0.192 | 0.194 | 0.368 | 0.108 |

*partially destroyed during acid hydrolysis

“-“ denotes that the values were below the quantification limit

**Supplementary Table 8**: Measured and predicted total amino acid content of the selected combinations. The ratios higher than 1 are presented here in bold.

| Amino acids (g/100g_product_) | Measured | Predicted | Measured/predicted |
| --- | --- | --- | --- |
| MOB1·HOB7 | 4.45 | 4.64 | 0.96 |
| MOB4·HOB13 | 4.53 | 3.37 | **1.35** |
| MOB5·HOB16 | 2.16 | 2.23 | 0.97 |
| MOB6·HOB15 | 6.85 | 4.76 | **1.44** |
| MOB6·HOB16 | 9.93 | 2.15 | **4.61** |
| MOB6·CNEC | 14.46 | 6.07 | **2.38** |
| MOB6·XAUT | 4.21 | 6.11 | 0.69 |
| MOB8·HOB13 | 14.02 | 2.31 | **6.06** |
| MOB8·HOB15 | 5.43 | 5.31 | 1.02 |
| MOB8·HOB18 | 4.20 | 7.49 | 0.56 |

**Supplementary Table 9**: Amount of various foodstuff needed to cover the daily essential amino acid (EAA) requirements of a 62 kg person (Walpole et al., 2012), as established by WHO/FAO/UNU, (2007). Abbreviations: WW = wet weight; AA = amino acid

| Amino acid | Requirements for 62 kg adults (g per day)^*^ | Quantity needed to meet individual AA requirements (g_WW_) | | | |
| --- | --- | --- | --- | --- | --- |
|  |  | **Whole egg** | **Raw chicken** | **Soybean** | **Tofu** |
| Histidine | 0.62 | 193 | 116 | 67 | 306 |
| Lysine | 1.86 | 200 | 118 | 84 | 395 |
| Threonine | 0.93 | 198 | 191 | 121 | 421 |
| Valine | 1.61 | 218 | 157 | 86 | 379 |
| Leucine | 2.42 | 188 | 148 | 101 | 333 |
| Isoleucine | 1.24 | 193 | 116 | 67 | 306 |
|  | | **Quantity needed to meet all AA requirements (g_WW_)** | | | |
|  |  | **218** | **191** | **121** | **421** |
| Limiting AA |  | Leucine | Valine | Valine | Valine |
| Reference | (WHO/FAO/UNU, 2007). | (Food Surveys Research Group (FSRG), 2006) | (Kim et al., 2017) | (Wang and Cavins, 1989) | (Wang and Cavins, 1989) |

* The average weight of a person is 62 kg (Walpole et al., 2012)

**Supplementary Table 10**: Amount of microbial biomass needed to cover the daily essential amino acid (EAA) requirements of a 62 kg person (Walpole et al., 2012), as established by WHO/FAO/UNU, (2007). The amino acid content of microbial protein is calculated taking into account 5% of moisture in the final product (Chemical Composition 1 - Unibio). MOB 5 and MOB 6 were not included due to poor data quality. Abbreviations: WW = wet weight; AA = amino acid

|  | Quantity of microbial biomass needed to meet individual AA requirements of a 62 kg person (g_WW_) | | | | | Quantity of microbial biomass needed to meet all AA requirements (g_WW_) | Limiting amino acid |
| --- | --- | --- | --- | --- | --- | --- | --- |
|  | **Histidine** | **Lysine** | **Valine** | **Leucine** | **Isoleucine** |  |  |
| MOB1·HOB7 | 243 | 272 | 321 | 447 | 523 | 523 | Isoleucine |
| MOB4·HOB13 | 233 | 472 | 255 | 398 | 398 | 472 | Lysine |
| MOB5·HOB16 | 194 | 763 | 669 | 941 | 1,140 | 1,140 | Isoleucine |
| MOB6·HOB15 | 190 | 408 | 233 | 235 | 234 | 408 | Lysine |
| MOB6·HOB16 | 177 | 179 | 159 | 156 | 150 | 179 | Lysine |
| MOB6·CNEC | 137 | 139 | 91 | 104 | 105 | 139 | Lysine |
| MOB6·XAUT | 265 | 489 | 244 | 324 | 289 | 489 | Lysine |
| MOB8·HOB13 | 162 | 153 | 118 | 128 | 126 | 162 | Histidine |
| MOB8·HOB15 | 212 | 332 | 215 | 313 | 265 | 332 | Lysine |
| MOB8·HOB18 | 204 | 261 | 297 | 369 | 301 | 369 | Leucine |
| MOB1 | 244 | 234 | 191 | 214 | 202 | 244 | Histidine |
| MOB4 | 278 | 582 | 299 | 478 | 359 | 582 | Lysine |
| MOB8 | 304 | 685 | 695 | 1,481 | 761 | 1,481 | Leucine |
| HOB7 | 63 | 338 | 1093 | 1,480 | 1,982 | 1,982 | Isoleucine |
| HOB13 | 62 | 325 | 752 | 866 | 1,049 | 1,049 | Isoleucine |
| HOB15 | 89 | 192 | 183 | 183 | 194 | 194 | Isoleucine |
| HOB16 | 91 | 387 | 525 | 504 | 488 | 525 | Valine |
| HOB18 | 79 | 115 | 151 | 110 | 128 | 151 | Valine |
| CNEC | 87 | 133 | 136 | 135 | 147 | 147 | Isoleucine |
| XAUT | 85 | 169 | 197 | 132 | 143 | 197 | Valine |

**Supplementary Table 11**: Stoichiometric calculations of microbial protein (MP) production using co-cultures of MOB and HOB. The inputs considered are biogas produced *via* anaerobic digestion, hydrogen and oxygen produced *via* water electrolysis and air.

| Inputs (mol) | | | | | | | Outputs (mol) | | | | | | | |
| --- | --- | --- | --- | --- | --- | --- | --- | --- | --- | --- | --- | --- | --- | --- |
| Water electrolysis | | | **Anaerobic digestion** | | **Air** | | **Aerobic MP production** | | | | | | | |
| H_2_O | **H_2_** | **O_2_** | **CH_4_** | **CO_2_** | **O_2_** | **N_2_** | **CH_4_** | **CO_2_** | **H_2_** | **O_2_** | **N_2_** | **MOB biomass** | **HOB biomass** | **H_2_O** |
| No air supplementation | | | | | | | | | | | | | | |
| 4.0 | 4.0 | 2.0 | 0.69 | 0.46 | - | - | 0.0 | 0.72 | 1.84 | 0.0 | - | 0.36 | 0.075 | 3.3 |
| With air supplementation | | | | | | | | | | | | | | |
| 4.0 | 4.0 | 2.0 | 0.69 | 0.46 | 0.86 | 3.2 | 0.0 | 0.65 | 0.0 | 0.0 | 3.2 | 0.36 | 0.14 | 5.1 |

# References

American Egg Board (2021). Nutrient Composition Tables. Available at: https://www.incredibleegg.org/professionals/manufacturers/technical-resources/nutrient-composition-tables [Accessed August 5, 2021].

Chemical Composition 1 - Unibio Available at: https://www.unibio.dk/end-product/chemical-composition-1/ [Accessed November 2, 2019].

De Rudder, C., Calatayud Arroyo, M., Lebeer, S., and Van de Wiele, T. (2020). Dual and Triple Epithelial Coculture Model Systems with Donor-Derived Microbiota and THP-1 Macrophages To Mimic Host-Microbe Interactions in the Human Sinonasal Cavities. *mSphere* 5. doi:10.1128/msphere.00916-19.

Ehsani, E. (2020). Diversity Management in the Synthetic and Enriched Microbial Communities.

Ehsani, E., Dumolin, C., Arends, J. B. A., Kerckhof, F.-M., Hu, X., Vandamme, P., et al. (2019). Enriched hydrogen-oxidizing microbiomes show a high diversity of co-existing hydrogen-oxidizing bacteria. *Appl. Microbiol. Biotechnol.* 103, 8241–8253. doi:10.1007/s00253-019-10082-z.

Food Surveys Research Group (FSRG) (2006). Food and Nutrient Database for Dietary Studies, 2.0. Available at: https://www.ars.usda.gov/northeast-area/beltsville-md-bhnrc/beltsville-human-nutrition-research-center/food-surveys-research-group/docs/fndds/ [Accessed October 31, 2019].

Hu, X., Kerckhof, F. M., Ghesquière, J., Bernaerts, K., Boeckx, P., Clauwaert, P., et al. (2020). Microbial Protein out of Thin Air: Fixation of Nitrogen Gas by an Autotrophic Hydrogen-Oxidizing Bacterial Enrichment. *Environ. Sci. Technol.* 54, 3609–3617. doi:10.1021/acs.est.9b06755.

Ishizaki, A., and Tanaka, K. (1990). Batch culture of *Alcaligenes eutrophus* ATCC 17697^T^ using recycled gas closed circuit culture system. *J. Ferment. Bioeng.* 69, 170–174. doi:10.1016/0922-338X(90)90041-T.

Khmelenina, V. N., Colin Murrell, J., Smith, T. J., and Trotsenko, Y. A. (2018). “Physiology and Biochemistry of the Aerobic Methanotrophs,” in *Aerobic Utilization of Hydrocarbons, Oils and Lipids* (Springer International Publishing), 1–25. doi:10.1007/978-3-319-39782-5_4-1.

Kim, H., Do, H. W., and Chung, H. (2017). A comparison of the essential amino acid content and the retention rate by chicken part according to different cooking methods. *Korean J. Food Sci. Anim. Resour.* 37, 626–634. doi:10.5851/kosfa.2017.37.5.626.

Letunic, I., and Bork, P. (2021). Interactive Tree Of Life (iTOL) v5: an online tool for phylogenetic tree display and annotation. *Nucleic Acids Res.*, 1–4. doi:10.1093/nar/gkab301.

Nielsen, J., and Villadsen, J. (1994). *Bioreaction Engineering Principles*. Springer US doi:10.1007/978-1-4757-4645-7.

Pruesse, E., Peplies, J., and Glöckner, F. O. (2012). SINA: Accurate high-throughput multiple sequence alignment of ribosomal RNA genes. *Bioinformatics* 28, 1823–1829. doi:10.1093/bioinformatics/bts252.

Raggi, T., Tacon, A. G. J., and Lemos, D. (2019). Feeding of juvenile cobia, Rachycentron canadum: Evaluation of practical feeds, comparison of commercial fishmeal replacers, and estimation of essential amino acid requirements. *J. World Aquac. Soc.* 50, 317–335. doi:10.1111/JWAS.12587.

Stamatakis, A. (2014). RAxML version 8: A tool for phylogenetic analysis and post-analysis of large phylogenies. *Bioinformatics* 30, 1312–1313. doi:10.1093/bioinformatics/btu033.

Vanhellemont, M., Baeten, L., and Verheyen, K. (2014). Relating changes in understorey diversity to environmental drivers in an ancient forest in northern Belgium. *Plant Ecol. Evol.* 147, 22–32. doi:10.5091/plecevo.2014.921.

Walpole, S. C., Prieto-Merino, D., Edwards, P., Cleland, J., Stevens, G., and Roberts, I. (2012). The weight of nations: An estimation of adult human biomass. *BMC Public Health* 12. doi:10.1186/1471-2458-12-439.

Wang, H., and Cavins, J. (1989). Yield and amino acid composition of fractions obtained during tofu production. *Cereal Chem.* 66, 359–361.

Weisburg, W. G., Barns, S. M., Pelletier, D. A., and Lane, D. J. (1991). 16S ribosomal DNA amplification for phylogenetic study. *J. Bacteriol.* doi:10.1128/jb.173.2.697-703.1991.

WHO/FAO/UNU (2007). Protein and amino acid requirements in human nutrition. Geneva, Switzerland Available at: www.who.int/bookorders [Accessed June 1, 2019].

Yu, J., Dow, A., and Pingali, S. (2013). The energy efficiency of carbon dioxide fixation by a hydrogen-oxidizing bacterium. *Int. J. Hydrogen Energy* 38, 8683–8690. doi:10.1016/j.ijhydene.2013.04.153.
